# Tumor‐Homing Phage Nanofibers for Nanozyme‐Enhanced Targeted Breast Cancer Therapy

**DOI:** 10.1002/adma.202403756

**Published:** 2024-09-05

**Authors:** Tao Yang, Qinglei Zhang, Yao Miao, Yang Lyu, Yajing Xu, Mingying Yang, Chuanbin Mao

**Affiliations:** ^1^ School of Materials Science & Engineering Zhejiang University Hangzhou Zhejiang 310027 P. R. China; ^2^ Department of Biomedical Engineering The Chinese University of Hong Kong Sha Tin Hong Kong SAR P. R. China; ^3^ Institute of Applied Bioresource Research College of Animal Science Zhejiang University Hangzhou Zhejiang 310058 P. R. China

**Keywords:** hydrogen peroxide decomposition, hypoxic tumor, phage nanofibers, photodynamic therapy, platinum nanozyme

## Abstract

Photodynamic therapy (PDT) eliminates cancer cells by converting endogenous oxygen into reactive oxygen species (ROS). However, its efficacy is significantly hindered by hypoxia in solid tumors. Hence, to engineer filamentous fd phage, a human‐friendly bacteria‐specific virus is proposed, into a nanozyme‐nucleating photosensitizer‐loaded tumor‐homing nanofiber for enhanced production of ROS in a hypoxic tumor. Specifically, Pt‐binding and tumor‐homing peptides are genetically displayed on the sidewall and tip of the fd phage, respectively. The Pt‐binding peptides induced nucleation and orientation of Pt nanozymes (PtNEs) on the sidewall of the phage. The resultant PtNE‐coated tumor‐homing phage exhibits significantly enhanced sustained catalytic conversion of hydrogen peroxide in hypoxic tumors into O_2_ for producing ROS needed for PDT, compared to non‐phage‐templated PtNE. Density functional theory (DFT) calculations verify the catalytic mechanism of the phage‐templated PtNE. After intravenous injection of the PtNE‐coated indocyanine green (ICG)‐loaded tumor‐homing phages into breast tumor‐bearing mice, the nanofibers home to the tumors and effectively inhibit tumor growth by the PtNE‐enhanced PDT. The nanofibers can also serve as a tumor‐homing imaging probe due to the fluorescence of ICG. This work demonstrates that filamentous phage, engineered to become tumor‐homing nanozyme‐nucleating tumor‐hypoxia‐relieving nanofibers, can act as cancer‐targeting nanozymes with improved catalytic performance for effective targeted PDT.

## Graphical abstract

Genetically engineered phages display a nanozyme‐nucleating peptide on the sidewall and a tumor‐homing peptide at the tip. They site‐specifically nucleate Pt nanozyme on the sidewall. The resultant phages can home to breast tumor and catalyze the conversion of tumoral hydrogen peroxide into oxygen. This strategy solves the problem of tumor hypoxia and improves the efficacy of photodynamic cancer therapy.

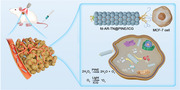

## 1 Introduction

It is well established that tumor cells exhibit accelerated metabolism, leading to poor lymphatic drainage and abnormal tumor vasculature.^[^ ^1^ ^]^ Consequently, during tumor progression, the areas of hypoxia develop within the tumor microenvironment (TME) due to compromised blood supply. Hypoxia has thus emerged as a characteristic feature of the TME in solid tumors.^[^ ^2^ ^]^ Moreover, cells in the TME typically experience higher levels of oxidative stress and hydrogen peroxide (H_2_O_2_) than healthy cells.^[^ ^3^ ^]^ The hypoxic characteristics of TME present a big challenge for some tumor therapy methods that rely on oxygen, such as photodynamic therapy (PDT). In a general PDT process, the photosensitizers absorb light and transfer the light energy to molecular oxygen, thereby generating the cytotoxic reactive oxygen species (ROS), such as singlet oxygen (^1^O_2_). Hypoxia within the tumor region results in a lack of oxygen, significantly reducing ROS generation and hindering the effectiveness of PDT treatment.

Nanozymes are artificial enzymes based on nanomaterials with intrinsic catalytic activities similar to natural enzymes. Due to their low cost and high catalytic stability, nanozymes show promise as substitutes for protein enzymes in numerous biomedical applications.^[^ ^4^ ^]^ Among these, platinum nanozymes (PtNEs) have been engineered to function as effective anticancer agents due to their ability to modulate TME.^[^ ^5^ ^]^ PtNEs exhibit superior resilience to harsh conditions within the TME compared to natural enzymes. Crucially, they efficiently catalyze the conversion of endogenous H_2_O_2_ into O_2_.^[^ ^6^ ^]^ Consequently, they are anticipated to synergize effectively with the O_2_‐dependent PDT process. However, the practical application of PtNEs for tumor therapy has been hindered by challenges including low colloidal stability and mass activity of platinum nanoparticles. The catalytic performance of PtNEs is greatly influenced by their intrinsic characteristics such as shape, size, and surface facets, yet the understanding of the structure‐function relationship in practical applications of PtNEs remains limited.^[^ ^7^ ^]^ Therefore, efforts should be directed toward developing novel platforms to enhance the stability and precisely control the catalytic properties of PtNEs during tumor therapy. Additionally, PtNEs do not naturally target tumors and thus concerns regarding biosafety and in vivo fate are limiting their applications.^[^ ^8^ ^]^ Although targeting ligands like peptides and antibodies have been engineered onto the surface of nanomaterials to enhance tumor uptake, the excessive addition of ligands may diminish the nanomaterial's ability to bind cell receptors.^[^ ^9^ ^]^ Moreover, the conjugation of ligands on the surface of nanomaterials could alter their catalytic properties, as the exposed surface is required to provide catalytic sites.

**Figure 1:**
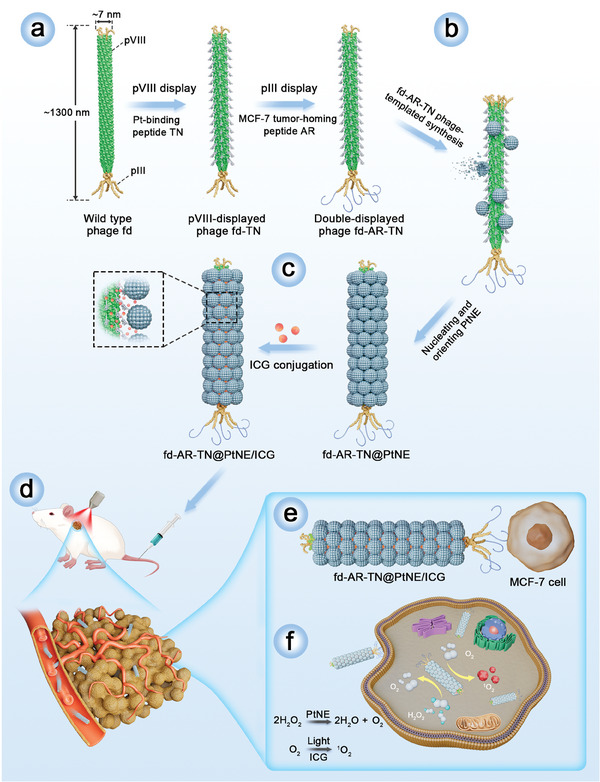
Schematic illustration of the synthesis of nanozyme (PtNE)‐coated tumor‐homing fd phage nanofibers using dual‐peptide‐displayed Pt‐binding tumor‐homing phage as a template and their use in nanozyme‐enhanced targeted breast cancer therapy. a) Genetic engineering of the fd phage nanofiber (≈1300 nm long, ≈7 nm wide). The Pt‐binding peptide TN was first fused to pVIII on the sidewall to form the pVIII‐displayed phage (fd‐TN). Then, the MCF‐7 tumor‐homing peptide AR was fused to pIII on the tip, generating the double‐displayed phage (fd‐AR‐TN). b) Phage‐templated nucleation of PtNEs on the sidewall. The Pt‐binding peptides displayed on pVIII facilitate the nucleation and oriented assembly of PtNEs on the sidewall of fd‐AR‐TN phage from a Pt precursor solution and the resultant PtNE‐coated tumor‐targeting phage is termed fd‐AR‐TN@PtNE. c) The conjugation of ICG photosensitizers onto the PtNE‐coated tumor‐targeting phage nanofibers. The ICG‐NHS ester molecules were covalently conjugated with the available −NH_2_ groups of the pVIII on the capsid of fd‐AR‐TN phages, forming ICG‐loaded PtNE‐coated tumor‐homing phage (fd‐AR‐TN@PtNE/ICG). d) The fd‐AR‐TN@PtNE/ICG phage nanofibers were intravenously injected into MCF‐7 tumor‐bearing mice to perform tumor therapy. e) The nanofibers could target the MCF‐7 tumor through the guidance of the AR peptide displayed on phage pIII. f) PtNEs on the sidewall of the nanofiber could catalyze H_2_O_2_ to decompose and generate O_2_ continuously. Consequently, the production of ROS was increased under the irradiation of NIR light for enhanced PDT of breast cancer by fd‐AR‐TN@PtNE/ICG.

Therefore, we propose to develop a tumor‐targeting PtNE‐nucleating phage nanofiber to overcome the challenges limiting the application of PtNEs in cancer therapy (**Figure** **1**). Filamentous fd phage is a human‐friendly virus that exclusively infects bacteria, serving as a natural and non‐toxic bionanofiber utilized in various biomedical applications from laboratory research to clinical practice.^[^ ^10^ ^]^ Comprising a single‐stranded DNA enclosed within a tubular capsid composed of five genetically modifiable coat proteins (including pIII and pVI at one end, pVII and pIX at the other end, and pVIII along the sidewall), fd phage offers a versatile platform for the genetic display of specific peptide ligands. Biopanning, a technique involving the interaction of a phage library with a target of interest followed by iterative rounds of enrichment, has been instrumental in discovering peptide ligands binding to inorganic materials, cells, or tissues.^[^ ^11^ ^]^ Using in vivo biopanning, we have successfully identified a breast tumor‐targeting peptide, AREYGTRFSLIGGYR (referred to as AR).^[^ ^12^ ^]^ Furthermore, the highly ordered structure of the pVIII (≈3900 copies) constituting the sidewall of filamentous phage makes the phage an ideal biological scaffold for controlling the nucleation of various nanomaterials.^[^ ^13^ ^]^ Material‐binding peptides displayed on helical pVIII proteins can interact with diverse nanomaterial precursors and direct the nucleation and orientation of the material in the form of nanocrystals.^[^ ^13b^ ^]^

Thus, to create tumor‐targeting PtNE‐nucleating phages, we displayed the AR peptide (discovered by us through in vivo biopanning^[^ ^12^ ^]^) and a reported Pt‐binding peptide (TLTTLTN, termed TN, identified against {100} plane through in vitro biopanning^[^ ^14^ ^]^) on the tip (pIII, 5 copies) and sidewall (pVIII, ≈3900 copies) of fd phage, respectively (Figure 1). The resulting double‐displayed fd phage variants were referred to as fd‐AR‐TN (Figure 1a). Subsequently, we utilized the double‐displayed phages as biological templates for PtNE nucleation on the sidewall, generating PtNE‐coated phage denoted as fd‐AR‐TN@PtNE (Figure 1b). We observed that TN peptides induced the nucleation of PtNEs with {100} plane exposed. The fd‐AR‐TN@PtNE phage nanofibers demonstrated efficient catalytic activity in decomposing H_2_O_2_ into O_2_. Due to the unique structures of PtNEs, their catalytic activities were improved in fd‐AR‐TN@PtNE, which were elucidated further through first‐principle calculations. We propose to enhance O_2_‐dependent breast cancer therapy using these phage‐templated PtNEs in conjunction with indocyanine green (ICG)‐based PDT as a model system. ICG, an excellent photosensitizer for PDT under near‐infrared (NIR) light irradiation, is widely used in NIR fluorescence imaging in clinical settings.^[^ ^15^ ^]^ Additionally, ICG can facilitate thermal imaging to guide tumor treatment via photothermal therapy when sufficient heat is generated to eradicate tumor cells.^[^ ^16^ ^]^ By chemically conjugating ICG onto the fd phage capsid, exploiting its high density and exposed amino sites, we created ICG‐modified fd‐AR‐TN@PtNE phage nanofibers (fd‐AR‐TN@PtNE/ICG) for injection into MCF‐7 tumor‐bearing mice via tail vein. The nanofibers homed to breast tumors guided by the AR peptides at the phage tip (Figure 1d,e). PtNEs on the sidewall alleviated hypoxia in the TME by catalyzing the decomposition of endogenous H_2_O_2_ to produce O_2_. Upon NIR light exposure, ICG converted O_2_ into ROS, enhancing PDT efficacy against MCF‐7 breast cancer (Figure 1f). This study shows that exploiting genetic engineering of fd phage to develop tumor‐targeting‐phage‐templated PtNEs, with significantly higher catalytic activities than the non‐phage‐templated PtNEs, can achieve enhanced targeted cancer therapy.

## 2 Results and Discussion

### 2.1 Synthesis and Characterization of fd‐AR‐TN@PtNE

We started with the genetic modification of fd phage by inserting the nucleotide sequences of the Pt‐binding peptides, TN, into the fd *gene VIII*, thereby fusing the peptides to the N‐terminus of the pVIII proteins, following our established protocol.^[^ ^12a^ ^]^ In an aqueous solution, these modified phage nanofibers (referred to fd‐TN) interacted with Pt ion precursors, guiding the nucleation and alignment of PtNEs. Subsequently, to develop tumor‐targeting fd phage, the breast cancer homing AR peptide was genetically fused to the exposed terminus of the pIII located at the distal end of fd‐TN phage, producing fd‐AR‐TN. The successful peptide display was verified through DNA sequencing of the resulting double‐displayed fd phage (Figure S1, Supporting Information). Subsequently, the fd‐AR‐TN phage nanofibers were mass‐produced using a flask‐shaking method by infecting their host bacteria *Escherichia coli*. Transmission electron microscopy (TEM) analysis with negative staining confirmed the morphology of the phage nanofibers (Figure S2, Supporting Information). Based on the TEM imaging results, fd‐AR‐TN phage nanofibers were estimated to be ≈1300 nm long and 7 nm wide. It should be noted that the length of the engineered phage was proportional to the viral genome packaged in the filamentous phage vector.^[^ ^17^ ^]^

**Figure 2:**
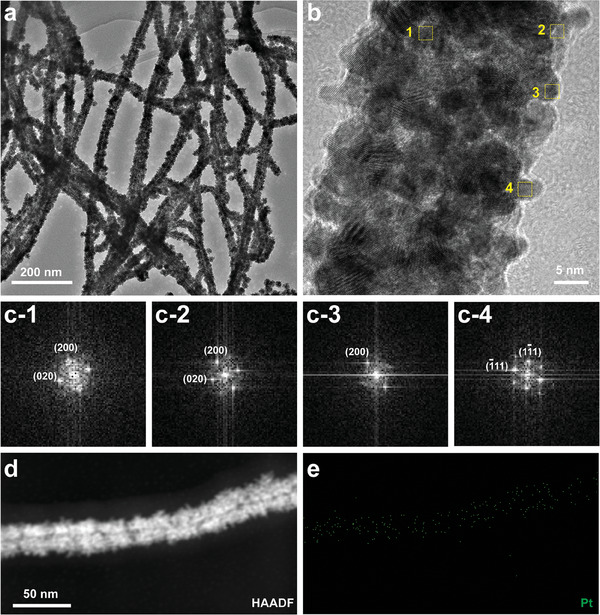
Verification of peptide‐mediated Pt nanozyme nucleation on the sidewall of double‐displayed fd phage. a) TEM images showing the morphology of the fd‐AR‐TN@PtNE nanofibers. b) High‐resolution transmission electron microscopy (HRTEM) lattice image of a straight region on the fd‐AR‐TN@PtNE nanofiber, indicating the close‐packed Pt nanocrystal morphology. Four typical areas (yellow rectangles) were selected to perform the Fast Fourier transformation (FFT). c) FFT of the selected nanocrystals in the HRTEM lattice image. The numbers (c‐1, c‐2, c‐3, c‐4) correspond to the rectangles (1, 2, 3, 4) in (b) one by one. d) High‐angle annular dark field scanning TEM (HAADF‐STEM) images of fd‐AR‐TN@PtNE nanofiber and e) the corresponding energy‐dispersive X‐ray (EDX) mapping of Pt element.

To achieve phage‐templated nucleation of PtNE to form fd‐AR‐TN@PtNE, the chloroplatinic acid precursor was incubated with fd‐AR‐TN phage at room temperature, followed by reduction using ascorbic acid and sodium borohydride agents at 0 °C (refer to Experimental Section for detailed synthesis procedures). The resulting PtNE‐coated phage nanofibers (fd‐AR‐TN@PtNE) were imaged by TEM without negative staining (**Figure** **2**a), confirming the nucleation and assembly of Pt nanocrystals along the length of fd‐AR‐TN. The templating effect of the fd phage was further evidenced by a concentration‐dependent synthesis of PtNEs (Figure S3, Supporting Information). Specifically, a low starting concentration of fd phage resulted in a significant portion of spontaneous non‐phage‐templated nucleation of Pt nanoparticles without stable binding to phage nanofibers (Figure S3a, Supporting Information). Doubling the concentration of the phage enabled the synthesis of phage‐templated PtNEs with almost no dispersed small particles, as observed under TEM imaging (Figure S3b, Supporting Information). However, further increasing the phage concentration caused noticeable colloidal instability, potentially leading to the aggregation of PtNE‐coated phage nanofibers (Figure S3c, Supporting Information). Therefore, we choose 0.04 mg mL^−1^ as the phage concentration and 2 mg mL^−1^ as the precursor concentration to produce the PtNE‐coated phage nanofibers in this study.

Pt nanocrystals exhibited a high degree of orientation around the sidewall of the phage nanofibers, owing to the binding affinity of TN peptides with Pt. The aligned PtNEs, ranging in size from 3 to 5 nm, were well‐crystallized, closely packed, and appeared to overlap, as indicated by the high‐resolution transmission electron microscopy (HRTEM) lattice image (Figure 2b). We randomly selected four areas from the lattice image and conducted Fast Fourier transformation (FFT). The resulting patterns exhibited a significant selectivity of enclosed crystal planes (Figure 2c); {100} diffracting facets were found to be predominant among the Pt nanocrystals on the nanofibers. Thus, as expected, these facet‐specific oriented nanocrystal growths were guided by the Pt‐{100} binding peptide (TN) displayed on phage capsids. HAADF‐STEM image and the corresponding EDX mapping further indicated a homogeneous distribution of PtNEs along the nanofiber (Figure 2d,e). Non‐phage‐templated PtNEs were also prepared as the control to our phage‐templated PtNEs (Figure S4, Supporting Information). The lattice image of the resulting PtNEs showed principally {111} diffracting facets among the Pt nanocrystals which was different from that in fd‐AR‐TN@PtNE. Collectively, we successfully synthesized PtNEs on the sidewall of fd‐AR‐TN@PtNE nanofibers with a preferred orientation of Pt‐{100} crystal planes.

**Figure 3:**
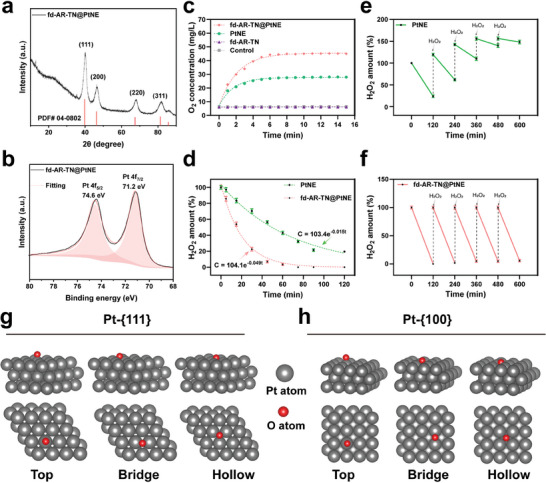
Structure and catalase‐like activities of fd‐AR‐TN@PtNE. a) Powder XRD pattern of fd‐AR‐TN@PtNE nanofiber. The nanofiber displays a typical face‐centered cubic phase of Pt (JCPDS PDF# 04–0802). b) Pt 4f XPS spectrum of fd‐AR‐TN@PtNE magnified from Figure S5 (Supporting Information). c) The enzymatic activity of PtNE and fd‐AR‐TN@PtNE to generate O_2_. The H_2_O_2_ solution was set as a control. d) Decomposition curve of H_2_O_2_ in the presence of PtNE or fd‐AR‐TN@PtNE. e,f) The cyclic catalytic activity of PtNE (e) and fd‐AR‐TN@PtNE (f) with repetitive supplement of H_2_O_2_ every 120 min. g,h) Side and top views of the possible adsorption sites (determined by DFT simulation), including the top, bridge, and hollow of Pt atoms, for chemisorption of O^*^ on Pt‐{111} (g) and Pt‐{100} (h). Red spheres are the oxygen atoms, and gray spheres are the Pt atoms.

The experimental synthesis of fd‐AR‐TN@PtNE nanofibers was scaled up to perform X‐ray diffraction (XRD) analysis (**Figure** **3**a). The sharp diffraction peaks in the patterns corresponded excellently with the phase of face‐centered cubic Pt (JCPDS PDF# 04–0802), confirming the successful synthesis of PtNEs with high crystallinity.^[^ ^18^ ^]^ The widening of the peaks observed at 20°–30°, indicating the presence of amorphous compositions, could be attributed to the phages from the nanofibers. X‐ray photoelectron spectroscopy (XPS) analysis revealed that fd‐AR‐TN@PtNE displayed Pt 4f peaks with a main valence of zero (Pt 4f7/2 at 71.2 eV and Pt 4f5/2 at 74.6 eV), originating from metallic PtNEs on the nanofibers (Figures 3b and S5, Supporting Information).^[^ ^19^ ^]^ The Pt contents in fd‐AR‐TN@PtNE (56.6 wt.%) were confirmed via inductively coupled plasma optical emission spectrometry (ICP‐OES) (Figure S6, Supporting Information).

### 2.2 Catalase‐Like Properties of fd‐AR‐TN@PtNE

Subsequently, the catalase‐like activities of fd‐AR‐TN@PtNE toward the decomposition of H_2_O_2_ were investigated. Catalase has the ability to decompose H_2_O_2_ into O_2_, leading to an enhanced level of dissolved oxygen and formation of gas bubbles in solutions containing H_2_O_2_ and catalase. Upon addition to the H_2_O_2_ solution, the fd‐AR‐TN@PtNE nanofibers exhibited enhanced catalase‐mimetic properties to generate dissolved oxygen (Figure 3c). Compared with the non‐phage‐templated PtNE group, the level of dissolved oxygen was significantly higher for the fd‐AR‐TN@PtNE group. The fd‐AR‐TN group showed no increased level of dissolved oxygen and produced no gas bubbles (Figure S7, Supporting Information), indicating that H_2_O_2_ was decomposed by the PtNEs on the phages. Furthermore, the amount of H_2_O_2_ consumed during the catalytic process was quantified by measuring the absorbance of H_2_O_2_ at ≈240 nm (Figure S8, Supporting Information). An enhanced catalytic activity was observed for fd‐AR‐TN@PtNE compared to PtNE under the same reaction conditions. The rate constant of the catalytic reaction was determined to be 0.049 min^−1^ for fd‐AR‐TN@PtNE and 0.015 min^−1^ for PtNE (Figure 3d). Importantly, the fd‐AR‐TN@PtNE nanofibers could maintain their catalytic activity in a TME‐mimetic acidic medium (pH = 6.5) after several catalytic cycles while the catalytic activity of PtNE was decreased quickly after only two catalytic cycles (Figure 3e,f). This could be attributed to the restriction of PtNE activity under acidic pH, whereas alkaline or neutral pH is commonly preferred by catalase‐like nanozymes.^[^ ^20^ ^]^ However, the fd‐AR‐TN@PtNE nanofibers can improve the catalytic activity directly by stabilizing the crystal plane with higher catalytic activity, thereby breaking this pH limitation. Continuous hypoxia alleviation in solid tumors during cancer treatment, such as PDT, presents a significant challenge.^[^ ^21^ ^]^ These data, particularly the stable catalytic activity after multiple cycles, suggest that our phage‐directed PtNE nanofibers provide promising platforms to address this issue and can enhance the efficacy of PDT for tumors.

A comprehensive understanding of the structural characteristics of PtNEs and their catalytic responses is essential for enhancing their applications. The mechanism of H_2_O_2_ decomposition on Pt nano‐catalysts has been proposed to involve a two‐step process.^[^ ^22^ ^]^ Initially, PtNEs react with one molecule of H_2_O_2_ to form the chemisorption of O^*^ on the Pt surface (referred to as Pt(O^*^)), releasing one molecule of water (H_2_O). Subsequently, another molecule of H_2_O_2_ continues to react with Pt(O^*^), restoring the metallic Pt surface and releasing one molecule of O_2_ and H_2_O. The rate‐limiting step of the reaction has been identified as the first step, involving the formation of chemisorption of O^*^ onto the surface of PtNEs.^[^ ^22^ ^]^ Surfaces favoring the chemisorption of O^*^ typically exhibit enhanced catalytic activity. To thoroughly understand the superior catalytic ability of fd‐AR‐TN@PtNE in our experiments, we conducted density functional theory (DFT) calculations to investigate the adsorption energy of the chemisorption of O^*^ on the Pt‐{111} and Pt‐{100} surfaces. Three different adsorption sites, including the top, bridge, and hollow sites on Pt‐{111} or Pt‐{100}, were considered for the binding configurations of Pt(O^*^) (Figure 3g,h). The chemisorption of O^*^ was found to preferentially bind to the hollow sites on Pt‐{111} and bridge sites on Pt‐{100) based on the calculated values. Table S2 (Supporting Information) summarizes all the calculated adsorption energies for the chemisorption of O^*^ on Pt‐{111} and Pt‐{100}. The adsorption energy of the chemisorption of O^*^ on Pt‐{100} (bridge site) is −1.61 eV, which is 0.5 eV stronger than its adsorption energy on Pt‐{111} (hollow site). These results indicate that the Pt‐{100} surface is more favorable for higher surface chemisorption of O^*^ than the Pt‐{111} surface. Indeed, this conclusion is also supported by the XPS results of fd‐AR‐TN@PtNE. The position of the Pt 4f peak for fd‐AR‐TN@PtNE is corresponding to its position for a higher level of surface oxidation on the Pt‐{100} lattice plane stabilized by the phage (Figure 3b). The experimental and computational findings presented here rationalize the higher catalase‐like activity of fd‐AR‐TN@PtNE (Figure 3c,d).

### 2.3 Preparation of fd‐AR‐TN@PtNE/ICG and Enhanced Photodynamic Reactions

**Figure 4:**
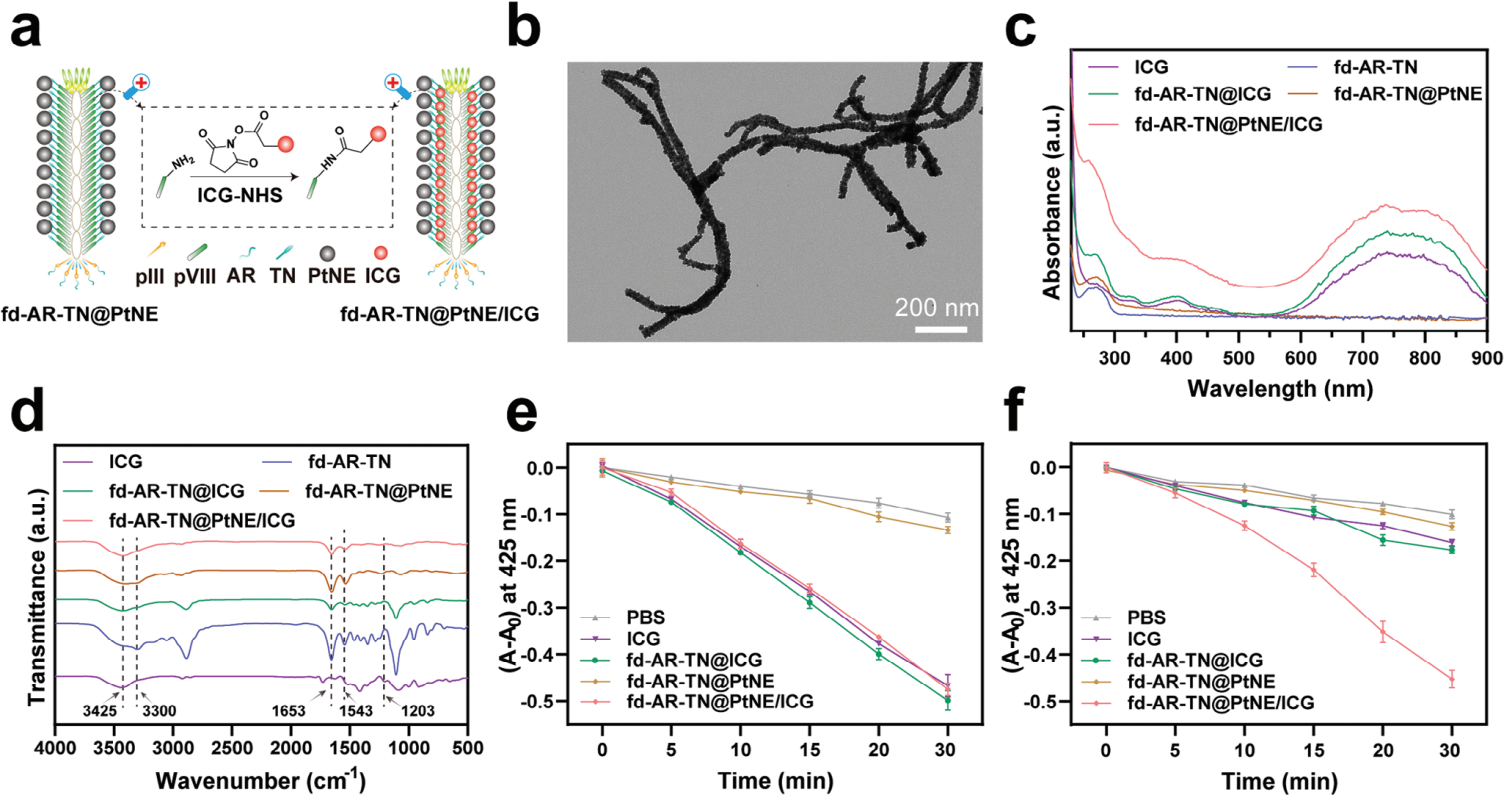
Morphology, absorption spectra, and property characterizations of ICG‐conjugated PtNE‐coated phage nanofibers (fd‐AR‐TN@PtNE/ICG). a) Schematic illustration of ICG conjugation onto the fd‐AR‐TN@PtNE nanofiber. The ICG‐NHS ester molecules were covalently conjugated with the available −NH_2_ groups of the pVIII on the capsid of fd‐AR‐TN phages, forming ICG‐loaded PtNE‐coated tumor‐homing phage (fd‐AR‐TN@PtNE/ICG). b) Representative TEM image of fd‐AR‐TN@PtNE/ICG nanofibers. c) Absorption and d) FTIR spectra of ICG, fd‐AR‐TN, fd‐AR‐TN@ICG, fd‐AR‐TN@PtNE and fd‐AR‐TN@PtNE/ICG. e,f) Photodegradation rates of the ^1^O_2_ indicator DPBF when incubated with PBS, ICG, fd‐AR‐TN@ICG, fd‐AR‐TN@PtNE and fd‐AR‐TN@PtNE/ICG in the presence of H_2_O_2_ under NIR light irradiation in air atmosphere (e) and N_2_ atmosphere (f).

Lysine residues and the N‐terminus of pVIII proteins provide abundant primary amine groups that serve as chemically modifiable sites for loading drugs, dyes, and photosensitizers onto phages.^[^ ^21^, ^23^ ^]^ Indocyanine green (ICG) was conjugated with PtNE‐coated phage nanofibers through the cross‐linking of N‐Hydroxysuccinimide ester (NHS ester) with the primary amine groups on the phages (**Figure** **4**a). The obtained ICG‐modified nanofibers (fd‐AR‐TN@PtNE/ICG) exhibited a well‐monodispersed morphology with PtNEs maintaining orientation along the length (Figure 4b). The absorption spectrum of the ICG‐modified nanofiber showed distinct absorption peaks in the NIR range corresponding to ICG, indicating the successful loading of the photosensitizer (Figure 4c). Further, the successful conjugation of ICG onto the phage capsid was analyzed by Fourier Transform infrared spectroscopy (FTIR) (Figure 4d). The absorption peaks at 3425 and 3300 cm^−1^ were derived from the stretching vibration of N‐H in amide bonds. Compared with the spectra of fd‐AR‐TN, the peak at 3300 cm^−1^ disappeared in the fd‐AR‐TN@PtNE/ICG, indicating the change of primary amides to secondary amides due to the reaction of ICG‐NHS with the primary amine groups of phages. The changes of peaks at 1653 cm^−1^ (stretching vibration of C═O) and 1543 cm^−1^ (rotation vibration of N─H) could also be assigned to the amido bond transition caused by the chemical crosslinking. Additionally, the characteristic NHS peak such as the stretching vibration of C─N─C (1203 cm^−1^) could hardly be found in the spectra of the fd‐AR‐TN@PtNE/ICG. These results confirmed that the loading of ICG was through chemical conjugation instead of physical adsorption. The amount of ICG on the nanofibers was determined by the calibration curve of absorbance at ≈785 nm, yielding a weight ratio of 5.6% (Figure S9, Supporting Information). The phage nanofibers coupled with the same ratio of ICG but without loading PtNEs were also prepared and termed fd‐AR‐TN@ICG. We also measured the photothermal effects of the nanofibers under NIR irradiation after the conjugation of ICG. The maximum temperature of fd‐AR‐TN@PtNE/ICG nanofibers depended on the concentration of ICG (Figures S10 and S11, Supporting Information). The photothermal responses of fd‐AR‐TN@ICG were similar to those of fd‐AR‐TN@PtNE/ICG nanofibers, indicating that temperature increases were primarily due to the photothermal effects of ICG (Figure S11, Supporting Information).

Subsequently, the ability of the fd‐AR‐TN@PtNE/ICG to generate ROS was investigated under NIR light irradiation (808 nm, 800 mW cm^−2^). Given that the ROS generated by ICG primarily comprises singlet oxygen (^1^O_2_), a typical ^1^O_2_ indicator, 1,3‐diphenylisobenzofuran (DPBF), was used to measure the ROS generation efficiency. The absorbance intensity of DPBF at ≈425 nm decreases upon ^1^O_2_ oxidation.^[^ ^24^ ^]^ A 100 µM H_2_O_2_ solution, with a concentration similar to that in the TME, was used for detection under normoxic and hypoxic conditions. The results indicated that the ^1^O_2_ generation ability of fd‐AR‐TN@PtNE/ICG was similar to that of fd‐AR‐TN@ICG under normoxic conditions (Figure 4e). However, improved ROS generation efficiency was observed for the fd‐AR‐TN@PtNE/ICG group under hypoxic conditions compared to the fd‐AR‐TN@ICG group (Figure 4f). These results suggest that fd‐AR‐TN@PtNE/ICG could effectively produce ROS via photodynamic reactions in the presence of H_2_O_2_ under tumor‐like hypoxic conditions. Consequently, the PDT efficiency of fd‐AR‐TN@PtNE/ICG could be significantly improved within a hypoxic TME with overexpressed H_2_O_2_ for enhanced targeted breast cancer therapy.

### 2.4 Tumor Cell Targeting and Cell Cytotoxicity In Vitro

**Figure 5:**
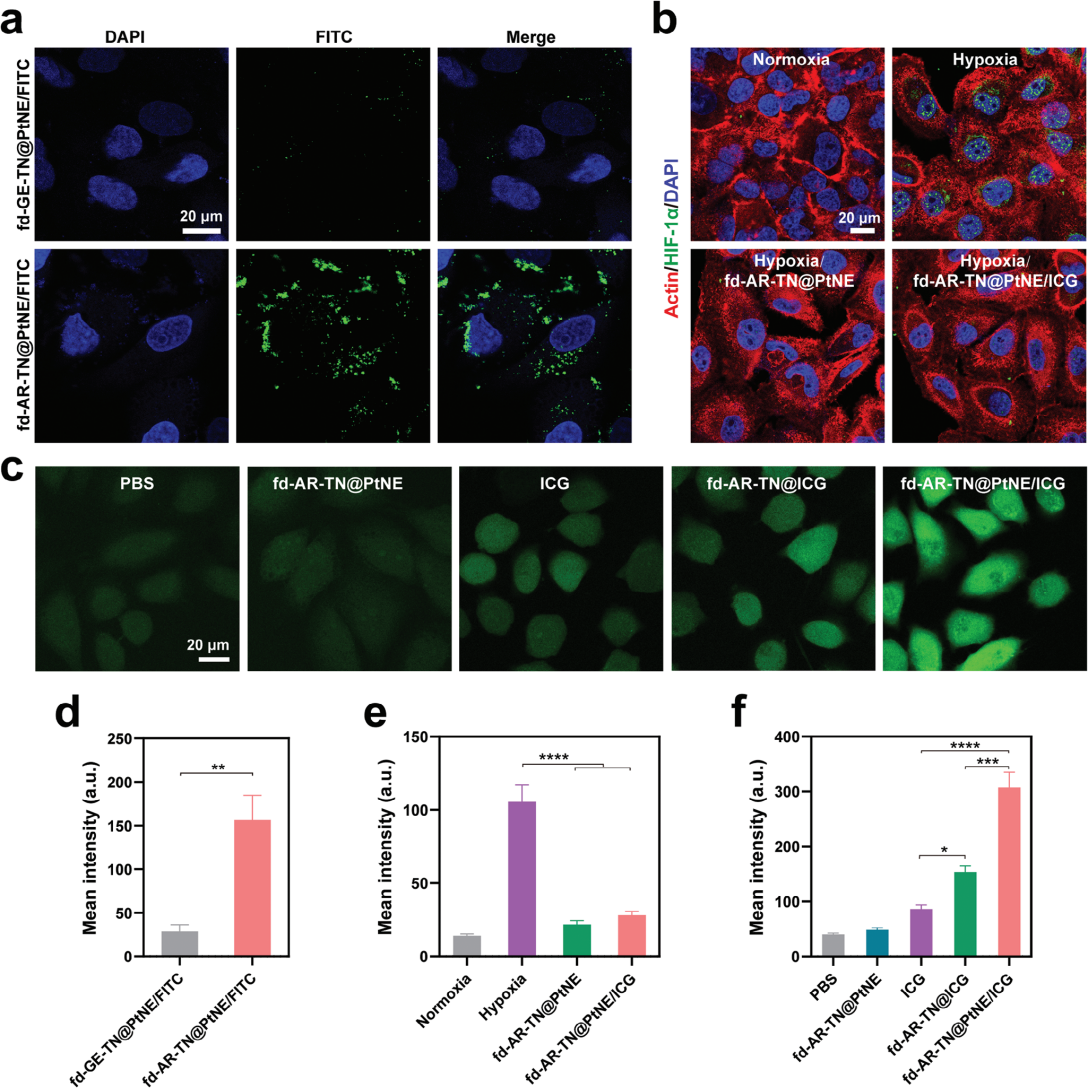
In vitro cellular uptake, suppression of hypoxic condition, and intracellular ROS generation. a) CLSM images of the MCF‐7 tumor cells co‐cultured with the fd‐GE‐TN@PtNE/FITC and fd‐AR‐TN@PtNE/FITC nanofibers. GE was a control peptide with a scrambled sequence of the peptide AR. All the nanofibers were conjugated with FITC to be conveniently observed under CLSM imaging. The cell nucleus was stained by DAPI (blue). b) HIF‐1α immunofluorescence staining (green) of MCF‐7 cells after various treatments indicated. Actin cytoskeleton was stained by fluorescent phalloidin conjugates (red). The cell nucleus was stained by DAPI (blue). c) ROS generation (green) in MCF‐7 cells treated by PBS, ICG, fd‐AR‐TN@ICG, fd‐AR‐TN@PtNE, and fd‐AR‐TN@PtNE/ICG in the hypoxic condition, as detected by DCFH‐DA, an intracellular ROS indicator. d) The relative intensity analysis of green fluorescence signals from FITC‐conjugated nanofibers of various groups in (a). ^**^ *p* < 0.01. e) The relative intensity analysis of green fluorescence signals from HIF‐1α immunofluorescence staining in (b). ^****^ *p* < 0.0001. f) The relative intensity analysis of green fluorescence signals from ROS staining in (c). ^*^ *p* < 0.05, ^***^ *p* < 0.001, ^****^ *p* < 0.0001.

The tumor‐homing ability of AR peptide‐engineered nanofibers was further confirmed by the selective uptake of MCF‐7 cells (**Figure** **5**a). To produce the control group (fd‐GE‐TN@PtNE), we replaced the tumor‐homing peptide AR with its scrambled sequence (GYFRSRLAIYTGRGE, termed GE) in the fd‐AR‐TN@PtNE phage. To facilitate the observation of the nanofibers under the confocal laser scanning microscope (CLSM), fluorescein isothiocyanate (FITC) molecules were conjugated onto the nanofibers through a reaction with the primary amine groups of the phages.^[^ ^25^ ^]^ The cells in the fd‐AR‐TN@PtNE/FITC group exhibited much stronger green fluorescence than those in the fd‐GE‐TN@PtNE/FITC group (Figure 5a). The fluorescence signals of the nanofibers were detected in both the cytoplasm and the cell nucleus. The quantification analysis of fluorescence intensity showed significant increases in the FITC fluorescence signals (green) in MCF‐7 cells in the fd‐AR‐TN@PtNE/FITC group compared to the fd‐GE‐TN@PtNE/FITC group (Figure 5d). These results revealed that the peptide AR displayed on the phage enabled the nanofibers to effectively target and bind to MCF‐7 tumor cells in vitro.

It has been widely recognized that the presence of hypoxia results in the intracellular production of hypoxia‐inducible factor (HIF)−1α protein.^[^ ^26^ ^]^ We then investigated the impact of PtNE‐coated phage nanofibers on the levels of HIF‐1α in hypoxic cells following incubation. As shown by immunofluorescence results in Figure 5b, no obvious HIF‐1α expression was observed in MCF‐7 cells with normoxia conditions. When exposed to hypoxic conditions, bright green fluorescence could be detected within tumor cells, indicating an increased level of HIF‐1α expression. After treatment with fd‐AR‐TN@PtNE or fd‐AR‐TN@PtNE/ICG, there was a very obvious decrease in the expression of HIF‐1α protein, indicating that the intracellular hypoxic environment was alleviated by the effective oxygenation facilitated by PtNE‐coated phage nanofibers (Figure 5e). The generation of intracellular ROS was detected during PDT treatment before the cell cytotoxicity evaluation. 2′,7′‐dichlorofluorescein diacetate (DCFH‐DA) was used as the detection probe, which produces green fluorescence when rapidly oxidized by ROS in tumor cells.^[^ ^27^ ^]^ MCF‐7 cells in the fd‐AR‐TN@PtNE/ICG group exhibited strong fluorescence after NIR light irradiation (Figure 5c,f), indicating intracellular ROS generation in large amounts. In contrast, the cells in other groups without PtNEs or ICG showed much weaker fluorescence under the hypoxic conditions (Figure 5c,f). In addition, the fd‐AR‐TN@ICG group displayed stronger fluorescence than ICG in MCF‐7 cells. This occurred as a result of a more extensive uptake of fd‐AR‐TN@ICG nanofibers than free ICG which was guided by the tumor‐targeting AR peptide at the tip of the phage (Figure S12, Supporting Information). These results indicate that the in vitro PDT efficacy was significantly enhanced by hypoxia relief using the fd‐AR‐TN@PtNE/ICG nanofibers.

**Figure 6:**
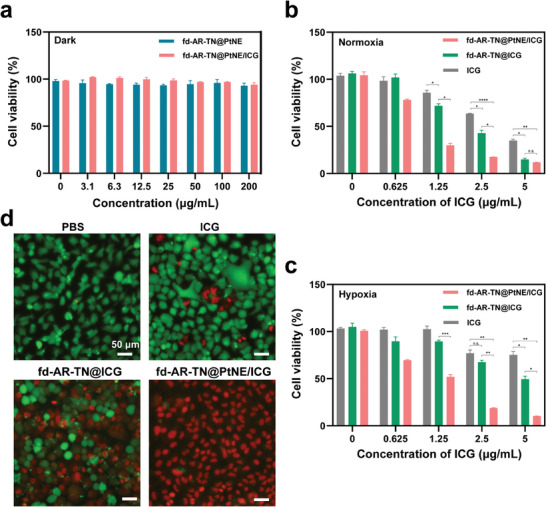
In vitro PDT efficacy of fd‐AR‐TN@PtNE/ICG on MCF‐7 tumor cells. a) Relative cell viability of MCF‐7 cells treated with the fd‐AR‐TN@PtNE or fd‐AR‐TN@PtNE/ICG nanofibers with different concentrations in the absence of NIR light irradiation. b,c) Relative cell viability of ICG, fd‐AR‐TN@ICG, and fd‐AR‐TN@PtNE/ICG treated MCF‐7 cells in normoxic (b) and hypoxic (c) conditions under NIR light irradiation. ^*^ *p* < 0.05, ^**^ *p* < 0.01, ^***^ *p* < 0.001, ^****^ *p* < 0.0001. d) Live‐dead staining of MCF‐7 cells treated by PBS, ICG, fd‐AR‐TN@ICG and fd‐AR‐TN@PtNE/ICG under NIR light irradiation in the hypoxic condition. Live cells (green) were stained by calcein‐AM and dead cells (red) were stained by propidium iodide.

The safety and PDT efficacy of fd‐AR‐TN@PtNE/ICG were assessed through an in vitro cytotoxicity assay against MCF‐7 cells using the cell counting kit‐8 (CCK‐8) approach. In the absence of NIR light irradiation, negligible cell cytotoxicity was observed even at a concentration of 200 µg mL^−1^ of the nanofibers, indicating the good biocompatibility of the fd‐AR‐TN@PtNE and fd‐AR‐TN@PtNE/ICG (**Figure** **6**a). The PDT efficacy of the nanofibers was evaluated with NIR light irradiation (808 nm, 800 mW cm^−2^) under both normoxic and hypoxic conditions. The fd‐AR‐TN@PtNE/ICG group exhibited no significant differences in cell cytotoxicity at the ICG concentration of 5 µg mL^−1^ compared to the fd‐AR‐TN@ICG group under the normoxic condition (Figure 6b). However, under the hypoxic condition (Figure 6c), the cytotoxicity against MCF‐7 cells of the fd‐AR‐TN@PtNE/ICG (≈90% cell death at an ICG concentration of 5 µg mL^−1^) was significantly higher than that of fd‐AR‐TN@ICG (≈50% cell death). It is worth noting that the cell cytotoxicity of the fd‐AR‐TN@PtNE/ICG group was also higher than the fd‐AR‐TN@ICG group under the normoxic condition at ICG concentrations of 0.625, 1.25, and 2.5 µg mL^−1^ (Figure 6b). This observation could be because the fd‐AR‐TN@PtNE/ICG group could immediately compensate for oxygen by PtNE‐catalyzed oxygen production while the fd‐AR‐TN@ICG group could not when the initial oxygen is consumed at the low ICG concentration, resulting in higher PDT efficiency of the former than the latter. The tumor cell killing ability was further demonstrated by live‐dead cell double staining. Calcein‐AM‐stained live cells emitted green fluorescence signals, while propidium iodide‐stained dead cells emitted red fluorescence signals. In the hypoxic condition, the treatment with PBS and free ICG resulted in relatively low red fluorescence (Figure 6d). With the treatment of fd‐AR‐TN@ICG, the percentage of dead MCF‐7 cells increased. Importantly, the fd‐AR‐TN@PtNE/ICG group exhibited the highest percentage of red fluorescence, indicating the most effective tumor cell killing efficacy, consistent with the CCK‐8 results.

### 2.5 Targeting MCF‐7 Tumor and Inhibiting Tumor Hypoxia In Vivo

The biocompatibility of the fd‐AR‐TN@PtNE/ICG nanofibers was further investigated. Hemolysis tests were conducted to assess the nanofibers' biocompatibility in blood (Figure S13, Supporting Information). The hemolysis percentages for both nanofibers were no more than 3.5%, even at an incubation concentration of 200 µg mL^−1^. Additionally, blood routine and biochemical analyses were performed for long‐term biosafety evaluation. The blood samples were collected at various time points after the fd‐AR‐TN@PtNE/ICG was administered. The blood routine analysis results showed that all nine common indices were within normal limits, indicating minimal blood toxicity of fd‐AR‐TN@PtNE/ICG at 16 days post‐injection (Figure S14, Supporting Information). Representative hepatic and kidney function markers for the blood biochemical analysis, including alanine transaminase (ALT), aspartate aminotransferase (AST), creatinine (CREA), blood urea nitrogen (BUN), uric acid (UA), and alkaline phosphatase (ALP), were measured (Figure S15, Supporting Information). Their levels, especially the AST levels, in the fd‐AR‐TN@PtNE/ICG group on days 8 and 16 were generally similar to those in the control group (untreated mice), suggesting the nanofibers’ liver and kidney compatibility. Overall, these results indicate the in vivo biocompatibility of the fd‐AR‐TN@PtNE/ICG phage nanofibers, enabling their further applications as theranostic materials.

**Figure 7:**
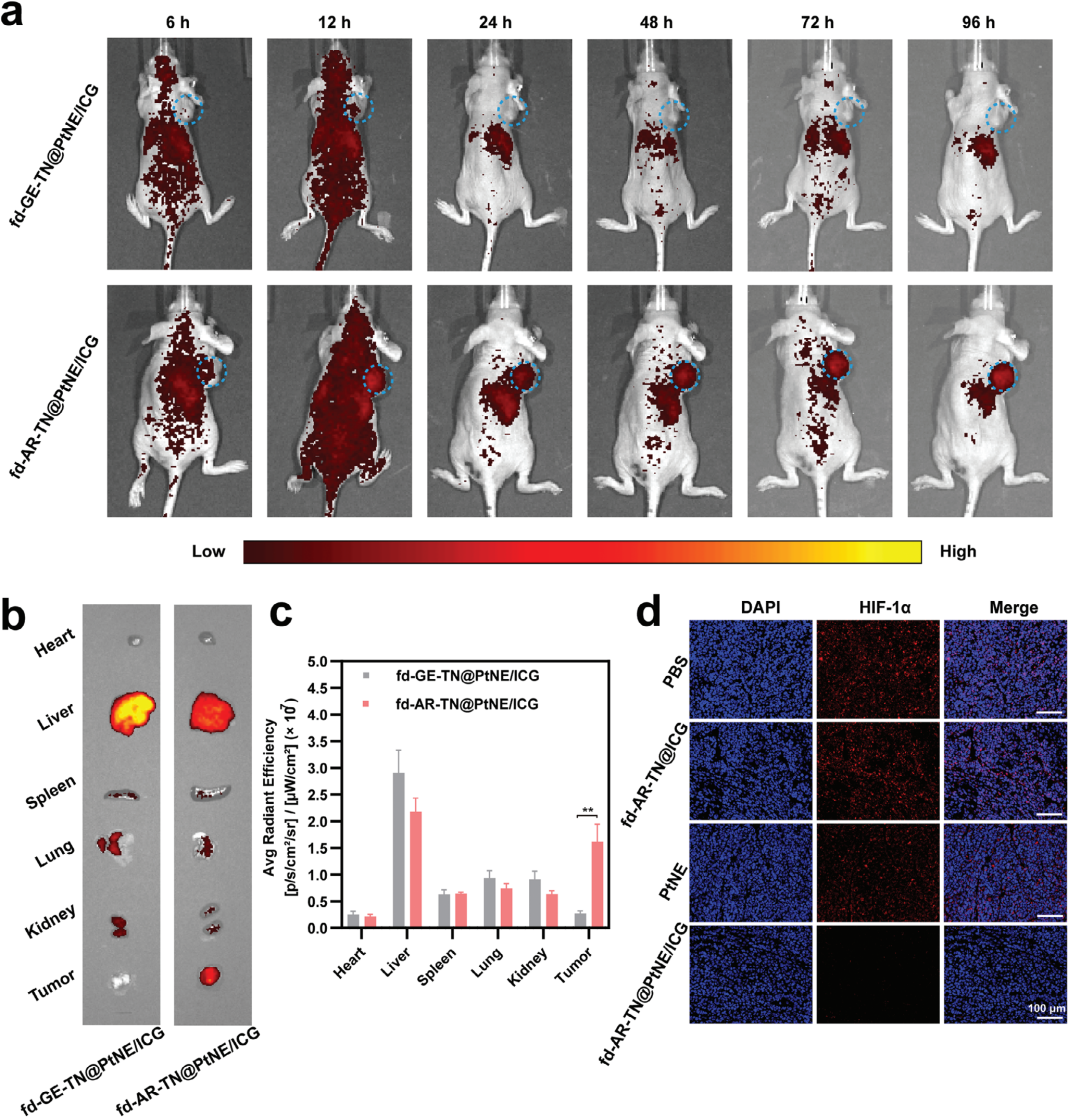
In vivo MCF‐7 tumor targeting ability of the phage nanofibers displaying the tumor‐homing peptide AR (vs the non‐tumor‐homing control peptide GE) at the tip and in vivo suppressing of tumor hypoxia by PtNE‐coated phage nanofibers. a) Fluorescence imaging of the fd‐GE‐TN@PtNE/ICG and fd‐AR‐TN@PtNE/ICG nanofibers distribution in the MCF‐7 tumor‐bearing mice. The images were obtained at 6, 12, 24, 48, 72 and 96 h post injection. The tumor sites were highlighted by blue circles. b) Fluorescence imaging of excised tumors and major organs (heart, liver, spleen, lungs, and kidney) 96 h post injection. c) Quantitative analysis of the mean ICG fluorescence intensity in the tissues excised after 96 h. ^**^ *p* < 0.01. d) HIF‐1α staining (red) of MCF‐7 tumor tissues 24 h post injection with the PBS, fd‐AR‐TN@ICG, PtNE and fd‐AR‐TN@PtNE/ICG. The cell nuclei were stained by DAPI (blue).

Then, we investigated the tumor‐homing ability of the nanofibers enabled by the peptide AR in vivo. The ICG conjugated to the nanofibers could not only generate ROS for PDT but also emit NIR fluorescence signals for optical imaging in vivo.^[^ ^28^ ^]^ We examined the imaging and biodistribution of the tumor‐homing fd‐AR‐TN@PtNE/ICG nanofibers in the MCF‐7 tumor‐bearing mice using an IR imaging system for small animals (**Figure** **7**a–c). The non‐homing fd‐GE‐TN@PtNE/ICG nanofibers were used as the control group. At 6 h after the intravenous injection, fluorescence from ICG was observed throughout the whole body, peaking at the tumor site at 12 h post‐injection (Figure 7a). Surprisingly, the high fluorescence intensity could still be observed at the tumor site of the fd‐AR‐TN@PtNE/ICG group after 96 h. In contrast, the fluorescence signal at the tumor site was hardly detectable at 96 h in the non‐tumor‐targeting fd‐GE‐TN@PtNE/ICG group. Furthermore, the fluorescence signals from the ex vivo organs also indicated the enhanced targeting ability of the fd‐AR‐TN@PtNE/ICG nanofibers against the MCF‐7 tumor tissues (Figure 7b). Quantitative analysis of fluorescence intensities in dissected tissues confirmed the significantly increased fluorescence signals of the fd‐AR‐TN@PtNE/ICG group compared to the fd‐GE‐TN@PtNE/ICG group (Figure 7c). Our results identified that the peptide AR could guide the nanofibers to home to MCF‐7 breast tumor tissues in vivo and accumulate at the tumor site for a relatively long period.

Furthermore, infrared thermal images of MCF‐7 tumor‐bearing mice were obtained using an IR thermal imaging camera to gather more precise information about the targeting ability of our tumor‐homing nanofibers at 12 h post‐injection. The surface temperature of the tumor tissues in the AR peptide‐engineered groups, including fd‐AR‐TN@ICG and fd‐AR‐TN@PtNE/ICG, increased to ≈43–44 °C with strong thermal signals (Figure S16, Supporting Information) after irradiation with NIR light (808 nm, 800 mW cm^−2^). Correspondingly, the PBS and free ICG groups only exhibited weak thermal signals, suggesting minimal heat generation. These results provide further evidence of the effective tumor accumulation of the tumor‐homing nanofibers mediated by the peptide AR after intravenous injection.

Inspired by the exceptional catalase‐like performance of the fd‐AR‐TN@PtNE/ICG in vitro, we conducted HIF‐1α immunofluorescent staining of tumor tissues to assess potential tumor hypoxia inhibiting properties of fd‐AR‐TN@PtNE/ICG in vivo. Solid tumors' hypoxic conditions can lead to HIF‐1α overexpression, which can be suppressed by O_2_ generation. Consequently, HIF‐1α levels correlate with O_2_ content, making HIF‐1α a typical hypoxia marker.^[^ ^29^ ^]^ As shown in Figure 7d, the tumor slices from the PBS group exhibited strong HIF‐1α immunofluorescence (red). In contrast, the tumor tissues injected with the fd‐AR‐TN@PtNE/ICG showed only weak immunofluorescence. The fd‐AR‐TN@ICG group also exhibited high expression of HIF‐1α fluorescence compared to the fd‐AR‐TN@PtNE/ICG group, suggesting that the PtNE on the fd‐AR‐TN@PtNE/ICG nanofibers alleviated the hypoxic microenvironment and suppressed the expression of HIF‐1α. However, the PtNE group without phage as a carrier expressed obviously more HIF‐1α immunofluorescence than the fd‐AR‐TN@PtNE/ICG group, further highlighting the importance of phage‐mediated targeted delivery. These results indicate the strong O_2_ generation ability of the fd‐AR‐TN@PtNE/ICG nanofibers in tumors, mitigating hypoxia by the PtNE‐mediated catalytic reaction in vivo. Subsequently, the increased tumor oxygenation could enhance PDT to restrict tumor growth in vivo.

### 2.6 Enhancing Tumor PDT In Vivo

**Figure 8:**
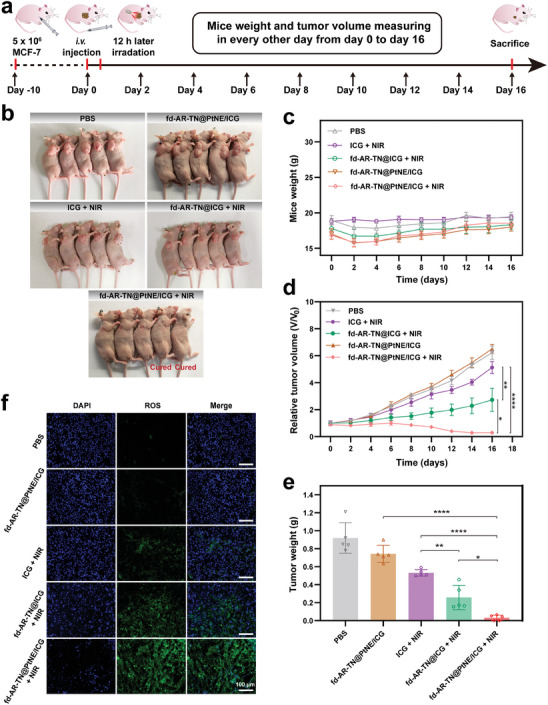
The targeted breast cancer therapy in vivo using the tumor‐homing nanofibers via nanozyme‐enhanced PDT in the MCF‐7 tumor‐bearing mice. a) Schematic illustration of the tumor therapy procedure in vivo. b) The actual photographs of the mice after the 16 days treatment. The tumor sites were highlighted by red circles. c) Body weights of the mice with different treatments (PBS, ICG + NIR, fd‐AR‐TN@ICG + NIR, fd‐AR‐TN@PtNE/ICG, and fd‐AR‐TN@PtNE/ICG + NIR, n = 5) during the 16 days of the therapy. + NIR, under NIR light irradiation. d) Relative tumor volume changes of MCF‐7 tumor‐bearing mice after various treatments. ^*^ *p* < 0.05, ^**^ *p* < 0.01, ^****^ *p* < 0.0001. e) The tumor weights acquired from the excised tumor tissues after various treatments. ^*^ *p* < 0.05, ^**^ *p* < 0.01, ^****^ *p* < 0.0001. f) ROS staining images of tumor tissues after various treatments indicated.

Given the high in vitro PDT efficacy, excellent tumor targeting, and good biocompatibility of our phage‐based nanofibers, we conducted in vivo PDT on the MCF‐7 tumor‐bearing mice. A total of 25 mice were randomly divided into five groups: PBS, fd‐AR‐TN@PtNE/ICG without NIR irradiation, ICG, fd‐AR‐TN@ICG, and fd‐AR‐TN@PtNE/ICG with NIR irradiation. For animals treated with irradiation, the NIR light (808 nm, 800 mWcm^−2^) was administered to the mice at 12 h post intravenous injection (**Figure** **8**a). The body weights of mice in all five groups showed a similar increasing trend, confirming the good safety of our phage‐based nanofibers in vivo (Figure 8c). The tumors of mice administered with fd‐AR‐TN@ICG were partially suppressed under the NIR light irradiation due to the phage‐mediated targeted delivery of ICG to the tumor sites (Figure 8d,e). However, the ICG group without the phage carrier showed only slight tumor growth inhibition due to the rapid drug metabolism of the free form. Excitingly, the results clearly indicated that tumor growth in the fd‐AR‐TN@PtNE/ICG group with NIR light irradiation was significantly inhibited. All tumors harvested from this group were very small, and 40% of mice were completely cured in the fd‐AR‐TN@PtNE/ICG + NIR group after the nanofiber administration for 16 days (Figure 8b). The therapeutic effects achieved by the fd‐AR‐TN@PtNE/ICG + NIR group were remarkable compared to the fd‐AR‐TN@ICG + NIR group, indicating synergistic effects resulting from increased O_2_ at the tumor sites for self‐sustained PDT. No tumor growth inhibition was observed for the fd‐AR‐TN@PtNE/ICG group without NIR light irradiation, further indicating that the fd‐AR‐TN@PtNE/ICG nanofibers had no noticeable dark toxicity. Additionally, the ROS production after PDT treatment in different groups was evaluated by the tumor tissue staining (Figure 8f). Compared with the fd‐AR‐TN@ICG + NIR group, the ROS signal was enhanced in the fd‐AR‐TN@PtNE/ICG + NIR group. Only a small amount of ROS signals was observed in the ICG + NIR group. The ROS staining results also confirmed the nanozyme‐enhanced targeted breast cancer therapy.

**Figure 9:**
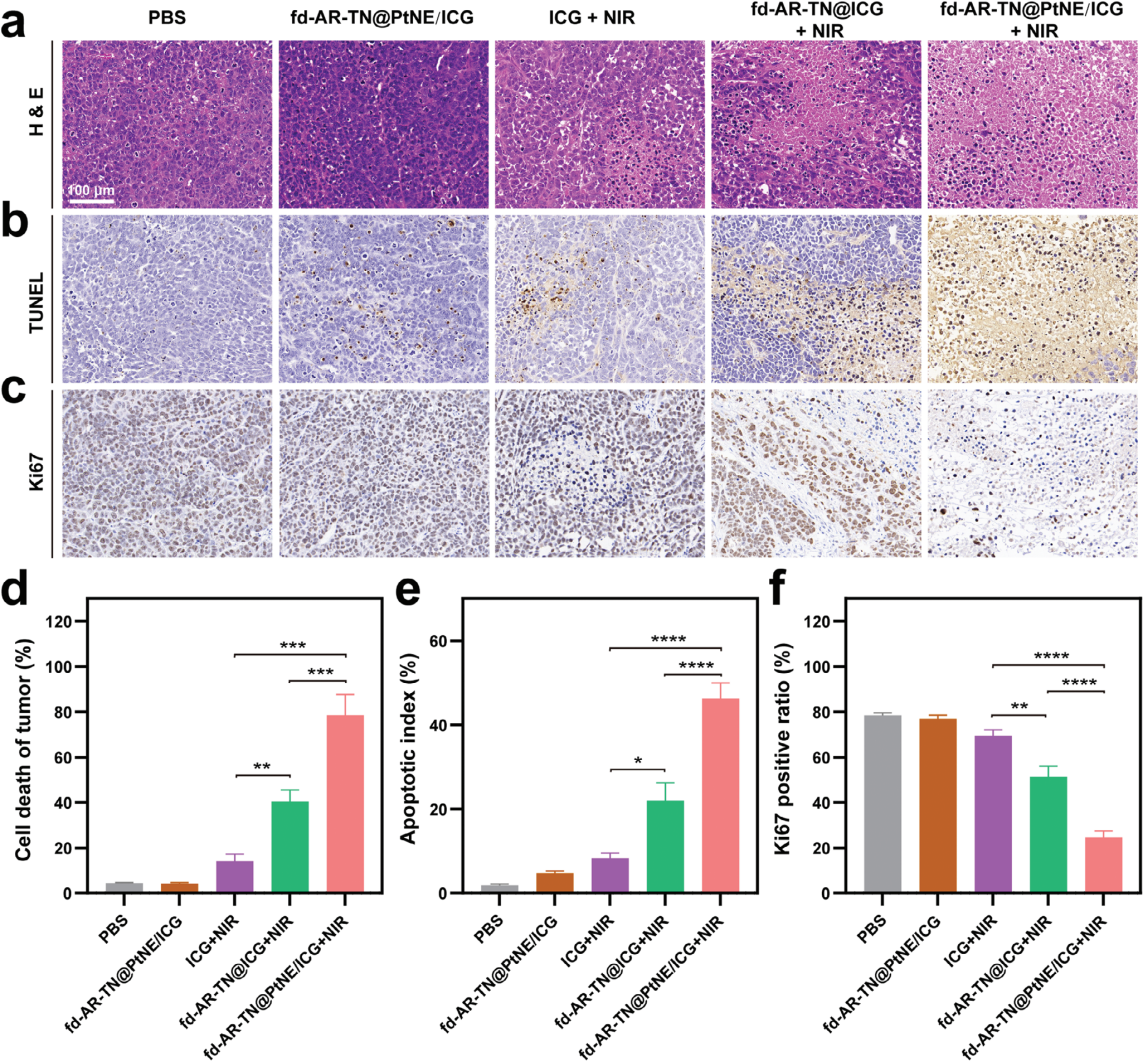
The histological and immunohistochemical evaluation of the tumors. a) H&E staining images of tumor slices from different groups, receiving treatments indicated. b) TUNEL and c) Ki67 immunohistochemical staining images of tumor tissues from various groups, receiving treatments indicated. d) Quantitative proportion of tumor cell death from the H&E staining images (n = 5 random respective sections). The apoptotic index and the Ki67 positive ratio were quantified in (e) and (f) from the immunohistochemical staining images (n = 5 random respective sections), respectively. ^*^ *p* < 0.05, ^**^ *p* < 0.01, ^***^ *p* < 0.001, ^****^ *p* < 0.0001.

The tumor slices from the various groups were then stained with hematoxylin and eosin (H&E) to assess their therapeutic efficacy (**Figure** **9**a,d). The mice administered with the fd‐AR‐TN@PtNE/ICG nanofibers under the NIR light irradiation exhibited the largest proportion of tumor cell death, confirming its high in vivo PDT efficacy. Furthermore, the TUNEL and Ki67 immunohistochemical staining were performed on the tumor slices to evaluate tumor cell apoptosis and proliferation levels, respectively. As expected, the fd‐AR‐TN@PtNE/ICG nanofiber group with NIR light irradiation showed the highest apoptotic index, indicating excellent tumor cell‐killing ability (Figure 9b,e). The minimum level of Ki67 staining signals was detected on the tumor slices of the fd‐AR‐TN@PtNE/ICG nanofiber group with NIR light irradiation, suggesting superior inhibition of tumor cell proliferation (Figure 9c,f), which was consistent with the results of H&E and TUNEL staining. Additionally, we collected slices of major organs including the heart, liver, spleen, lung, and kidney from the mice of all groups and performed H&E staining to study potential histological toxicity after tumor PDT (Figure S17, Supporting Information). Indeed, no obvious inflammation or pathological abnormalities were observed in the slices of any group.

## 3 Conclusion

In summary, we successfully synthesized PtNE‐coated tumor‐homing fd phage nanofibers and found that the nanofibers exhibited a combination of desired properties, including enhanced catalase‐like catalysis and outstanding tumor homing, leading to targeted breast cancer therapy. The overexpression of H_2_O_2_ molecules in solid tumors was catalyzed by PtNEs on the fd phage nanofibers to generate O_2_, which was further converted to ROS by ICG molecules upon NIR light irradiation. The continuous O_2_ generation from the catalytic reaction significantly improved PDT efficacy by alleviating the hypoxic tumor microenvironment. Importantly, the Pt‐binding peptides engineered on the phage's sidewall induced site‐specific nucleation and orientation of PtNEs, with the desired catalytic crystal surfaces stabilized by the peptide sequences. The tumor‐homing peptide AR engineered at the tip of the phage facilitated selective breast cancer targeting. By bearing PtNEs and photosensitizers on the sidewall and tumor‐targeting ligands on the distal end, on a single phage platform, the resultant phage nanofibers maximized the advantages of phage‐based biomaterials. These innovative nanoplatforms hold promise for effective anti‐tumor strategies.

## Conflict of Interest

The authors declare no conflict of interest.

## Supporting information

Supporting Information

## Data Availability

The data that support the findings of this study are available from the corresponding author upon reasonable request
