# Supplementary material for: Tumor‐Homing Phage Nanofibers for Nanozyme‐Enhanced Targeted Breast Cancer Therapy
Source: Adv Mater. 2024 Sep 5;37(2):2403756. doi: 10.1002/adma.202403756 (PMC11733710; doi:10.1002/adma.202403756)
Supplement: Supplementary file 1 — Supporting Information [file ADMA-37-2403756-s001.pdf]

ADVANCED MATERIALS

Supporting Information

for *Adv. Mater.*, DOI 10.1002/adma.202403756

Tumor-Homing Phage Nanofibers for Nanozyme-Enhanced Targeted Breast Cancer Therapy

*Tao Yang, Qinglei Zhang, Yao Miao, Yang Lyu, Yajing Xu, Mingying Yang and Chuanbin Mao**

Supporting Information

Experimental methods

Supporting Table S1-S2

Supporting Figure S1-S17

Experimental methods

Materials: Chloroplatinic acid hydrate ($H_2PtCl_6 \cdot xH_2O$), ascorbic acid and 1,3-diphenylisobenzofuran (DPBF) were obtained from Sigma-Aldrich. Hydrogen peroxide (H_2O_2) and sodium borohydride ($NaBH_4$) were obtained from Sinopharm Group Co. Ltd. *Pst*I, *Hind*III and *Bgl*I enzymes and T4 DNA ligase were obtained from New England Biolabs. Indocyanine Green NHS ester (ICG-NHS) was purchased from Duofluor Inc. Fluorescein isothiocyanate (FITC) was purchased from Aladdin. 2′,7′-dichlorofluorescin diacetate (DCFH-DA) was obtained from Solarbio Life Sciences. Ultrapure water (18.2 MΩ, Millpore) was used to prepare all aqueous solutions in all experiments.

Characterizations: Transmission electron microscopy (TEM) images were obtained using Hitachi JEM-1200EX with a 120 kV accelerating voltage. The negative staining was performed with 1% uranyl acetate. High-resolution transmission electron microscopy (HRTEM) lattice images were obtained from FEI Tecnai G2 F20 S-TWIN with a 200 kV accelerating voltage. X-ray photoelectron Spectroscopy (XPS) was measured by Thermo Scientific K-Alpha. X-ray diffraction (XRD) was measured on Rigaku SmartLab SE. The contents of Pt were measured by inductively coupled plasma optical emission spectrometry (ICP-OES, Agilent720ES). The absorption spectra were measured on a SpectraMax M2 instrument. Confocal Laser Scanning Microscope (CLSM) images were obtained from a Zeiss LSM 800 instrument. All the animal experiments were approved and guided by Animal Ethics Committee of Zhejiang University.

Genetic engineering of double-display phages: We used our fd388 double-display system derived from the fd-tet vector to generate the genetic engineered phage nanofibers.[1] The Pt-binding peptide (fused to the pVIII protein) and tumor-homing peptide (fused to the pIII protein) were engineered on a single phage nanofiber. Firstly, the fd388 vector was digested by *Pst*I and *Hind*III at 37 °C for 4 hours and purified via the agarose gel electrophoresis. Meanwhile, a pair of annealed oligonucleotides (Table S1) encoding for the Pt-binding peptide was mixed well with equimolar ratio and annealed (heated to 94 °C for 10 mins, and then cooled to 25 °C slowly) to get the double-stranded DNA fragments. The digested fd388 vector was then ligated with the annealed DNA fragments by the T4 DNA ligase (16 °C for 16 hours). The recombinant plasmid, p-fd-TN, was transferred into the JM109 competent cells. Then, p-fd-TN was digested by *Bgl*I at 37 °C for 4 hours and purified via the agarose gel electrophoresis. The annealed oligonucleotides (Table S1) encoding for the tumor-homing peptide AR were mixed with an equimolar ratio and annealed (heated to 94 °C for 10 min, and then cooled to 25 °C slowly) to get the double-stranded DNA fragments. The digested p-fd-TN plasmid was then ligated with the annealed DNA fragments by the T4 DNA ligase (16 °C for 16 hours). The recombinant plasmid p-fd-AR-TN was transferred into the JM109 competent cells. Positive clones were selected and sequenced to confirm the correct DNA insertion.

Generation of double-displayed phage nanofiber fd-AR-TN: The JM109 *Escherichia coli* (*E.coli*) transforming the recombinant plasmid was shaken in LB media with tetracycline (20 μg mL^{-1}) at 37 °C overnight. The culture was centrifuged (12000g, 4°C) for 20 minutes to remove the bacteria. The double-displayed phage in the supernatant was used to infect TG1 *E.coli* in the log phase and incubated with 37 °C for 30 minutes. The culture then was transferred into 1L LB media with tetracycline (20 μg mL^{-1}) in the 2L flask to amplify the double-displayed phage. The flask was shaken at 28 °C, 180 rpm for 18 hours. The mixture was centrifuged (12000g, 4°C) for 30 minutes to remove the bacteria and the supernatant was precipitated twice with 16.7% PEG 8000/2.5 M NaCl. The final phage precipitates were resuspended in ultrapure water and centrifuged

(18000g, 4°C) again to remove the residual bacteria. The final phage nanofiber solutions were dialyzed against ultrapure water for 48 hours and preserved at 4 °C.

Synthesis of fd-AR-TN@PtNE nanofibers: The double-displayed fd-AR-TN phage was mixed with aqueous H_2PtCl_6 solution (2 mg/mL Pt(IV)) and incubated for 8 h at 25 °C. The solution temperature was lowered to 0 °C and aqueous ascorbic acid solution (5 mM) was added and mixed well. Immediately, the freshly prepared aqueous $NaBH_4$ solution (0.1 mM) was injected into the solution to nucleate Pt nanocrystals homogeneously. The mixture was continuously aged at 0 °C for 2 hours. The fd-AR-TN@PtNE phage nanofibers were then separated by centrifugation (12000g, 4 °C) and rinsed with ultrapure water for 3 times. Non-phage-templated PtNEs were also synthesized as the control with a similar procedure except for the incubation of the phage and H_2PtCl_6 solution.

The Catalase-Like Activity of fd-AR-TN@PtNE nanofibers: The catalase-like activity for the catalytic decomposition of H_2O_2 was firstly evaluated by the generation of O_2 bubbles and the detection of dissolved oxygen level. The reaction was performed by mixing ultrapure water (Control), fd-AR-TN (1 μg mL^{-1}), free PtNE (1.5 μg mL^{-1}) or fd-AR-TN@PtNE (2.5 μg mL^{-1}) with H_2O_2 solutions (30 mM). After reaction lasted at 37 °C for 30 minutes, the photographs were taken. For quantifying the level of dissolved oxygen during the reaction, a real-time measurement was conducted using a DOS-1703 portable meter. The reaction was performed by mixing the samples aforementioned with 1 mM H_2O_2 solutions at room temperature for 15 minutes. The dissolved oxygen changes were recorded every 60 seconds by the portable meter.

Then, we determined the rate constant of the catalytic reaction according to the decomposition curves of H_2O_2 (30 mM) in the presence of fd-AR-TN@PtNE (2.5 μg mL^{-1}). The relative concentration of H_2O_2 was estimated by the absorbance at 240 nm. A series of catalytic reactions with different time points, including 5, 15, 30, 45, 60, 75, 90 and 120 min, were conducted at 37 °C. After reactions, the mixtures were centrifuged

immediately to remove the nanofibers, and the amount of the remaining H_2O_2 were measured by the absorbance spectra.

The catalytic durability of fd-AR-TN@PtNE was validated through the cyclic H_2O_2 decomposition reactions. The reaction was performed by mixing fd-AR-TN@PtNE (2.5 μg mL^{-1}) with H_2O_2 (30 mM) in PBS buffer under an acidic pH of 6.5 at 37 °C. The consumed H_2O_2 was repetitively added at the time points of 120, 240, 360, 480 and 600 min. At each time point just before the addition of the supplement of H_2O_2, the amount of the remaining H_2O_2 was determined by the absorption spectra.

Density Functional Theory (DFT): DFT calculations were performed via Vienna ab initio Simulation Package using the method of projector augment wave.[2] Generalized gradient approximation of the Perdew-Burke-Ernzerhof (PBE) function was the exchange-correlation function.[3] For the surface calculation, 2 × 2 × 1 K points were sampled from the Brillouin zone.[4] The cutoff energy was set to be 500 eV and the structure relaxation was conducted until convergence criteria of force (0.02 eV $Å^{-1}$) and energy (1×10^{-5} eV) were met. A vacuum layer (15 Å) was created to eliminate the interaction of the periodic structure from the surface model. Van der Waals interaction force was adjusted using a zero damping DFT-D3 approach of Grimme.[5] The surfaces of Pt(111) and Pt(100) were modeled via the slab geometry based on the periodically repeated (4 × 4) unit cell with three atomic layers. The experiment equilibrium Pt lattice constant has been determined to be 3.92 Å.[6] Adsorption energies are calculated to be the energies of the metal slab containing the adsorbate with respect to the energies of a clean slab and a free gas-phase adsorbate.

Preparation of fd-AR-TN@PtNE/ICG nanofibers: ICG succinimidyl easter (NHS easter) was dissolved in N,N-Dimethylformamide to prepare the stock solution (1 mg mL^{-1}). The fd-AR-TN@PtNE nanofibers were diluted in the PBS buffer (0.1 mg mL^{-1}). One milliliter of the ICG stock solution was added into nine milliliters of the nanofiber solution and then rocked gently for 6 hours at 25 °C. The conjugation product was

separated from the mixture by centrifugation and rinsed using PBS. The amount of ICG conjugated onto the fd-AR-TN@PtNE/ICG nanofibers was determined by the absorption spectra.

ROS generation of fd-AR-TN@PtNE/ICG nanofibers: The singlet oxygen indicator DPBF was used to probe the ROS generation. Typically, 10 μL of DPBF/ethanol solution was added into the fd-AR-TN@PtNE/ICG solution (100 μg mL^{-1}) with H_2O_2 (100 μM). The solution was irradiated with the 808 nm light (0.8 W cm^{-2}) for different time periods. The amount of ROS generation was measured via the absorption spectra of DPBF before and after the NIR light irradiation.

For the hypoxic condition, the DPBF and the nanofiber mixture solution were bubbled with N_2 for 20 minutes just before the addition of H_2O_2. Then, the solution was exposed to the NIR light for different time periods. The ROS generation was measured via the absorption spectra of DPBF before and after the NIR light exposure.

Photothermal effects: The fd-AR-TN@PtNE/ICG nanofiber solutions with various ICG concentrations (5, 10, and 15 μg mL^{-1}) were exposed to the 808 nm light (0.8 W cm^{-2}). The solution temperature changes were recorded every 15 seconds by the thermal camera.

In vitro MCF-7 cell targeting: MCF-7 cells obtained from American Type Culture Collection originally were cultured with the growth medium (DMEM with high glucose, 10% FBS) under 5% CO_2. The FITC was first conjugated to prepare the fd-AR-TN@PtNE/FITC nanofibers before the incubation with MCF-7 cells. Briefly, an FITC stock solution (1 mg mL^{-1}) was added into the fd-AR-TN@PtNE nanofibers in the sodium bicarbonate buffer and then rocked gently for 8 hours at 25 °C. The conjugation products were separated from the mixture by centrifugation and rinsed using PBS. Subsequently, the FITC conjugated nanofibers were incubated with MCF-7 cells at 37 °C for 4 hours. The cells were washed using the PBS buffer and then fixed with

paraformaldehyde. The cell nuclei were stained by DAPI and washed with PBS twice before the observation under CLSM. The non-targeting fd-GE-TN@PtNE/FITC group was used as the control group. Free FITC, fd-GE-TN@FITC and fd-AR-TN@FITC were also used to evaluate the selective uptake of MCF-7 cells in another independent experiment.

Intracellular HIF-1α immunofluorescence: MCF-7 cells were seeded in confocal dishes with a number of 5×10^4 cells per well. After different treatments, cells were fixed with 4% paraformaldehyde at room temperature for 15 minutes followed by permeabilizing with 0.3% Triton X-100 for 10 minutes and blocking with 1% BSA for 1 hour. Then, cells were incubated with rabbit anti-HIF-1α primary antibody overnight at 4 °C and stained with Alexa Fluor® 488-conjugated anti-rabbit IgG (H+L) secondary antibody for 1 hour at 25 °C. Finally, the actin cytoskeleton was stained using Alexa Fluor® 555-labeled phalloidin for 45 minutes and the cell nucleus was stained by DAPI for 5 minutes at room temperature.

Intracellular ROS generation: DCFH-DA was used to be the intracellular ROS indicator to measure the ROS generation in MCF-7 cells. The cells were treated by PBS, ICG, fd-AR-TN@ICG, fd-AR-TN@PtNE or fd-AR-TN@PtNE/ICG, which was diluted in the growth medium for 4 h at the hypoxic condition. The hypoxic condition in the incubator was achieved by replacing the gas supply of air with N_2. For the ICG-bearing groups, the equivalent concentration of ICG is 5 μg/mL. Afterwards, the sample-containing medium was replaced by the DCFH-DA working solution (1×10^{-5} M) and incubated with the cells at 37 °C for 30 minutes. The tumor cells were then irradiated with 808 nm light (0.8 W cm^{-2}) for 5 minutes. The ROS generation indicated by the green fluorescence with the excitation at 488 nm was observed through the CLSM.

In vitro PDT: MCF-7 cells were seeded in the 96-well microplate with the amount of 1×10^4 cells per well. For the biocompatibility evaluation, the tumor cells were co-

cultured with various concentrations of fd-AR-TN@PtNE or fd-AR-TN@PtNE/ICG nanofiber solutions (0, 3.1, 6.3, 12.5, 25, 50, 100, 200 μg mL^{-1}) for 24 hours. The cell viability was determined via Cell Counting Kit-8 (CCK-8). Generally, the culture medium containing nanofibers was replaced with CCK-8 reagents diluted in the growth medium. After incubation for 1 hour at the incubator, the absorbance intensity at 450 nm was detected. For the evaluation of the *in vitro* PDT efficacy, tumor cells were incubated with different concentrations of ICG, fd-AR-TN@ICG and fd-AR-TN@PtNE/ICG solutions (the concentration of ICG was 0, 0.625, 1.25, 2.5, 5 μg mL^{-1}) for 4 hours at the normoxic or hypoxic condition. The cells were washed with DMEM and then irradiated with 808 nm light (0.8 W cm^{-2}) for 5 minutes. After the further incubation in the growth medium for 20 hours, cell viability was detected via CCK-8 method.

Live/dead cell staining: MCF-7 cells were cultured in growth medium under the hypoxic condition for 24 hours at the incubator. The cells were then treated by each of the different groups (including PBS, ICG, fd-AR-TN@ICG, and fd-AR-TN@PtNE/ICG) that was diluted in the growth medium (the concentration of ICG was 5 μg mL^{-1}) for 4 hours. After which, the cells were exposed under an 808 nm light (0.8 W cm^{-2}) for 5 minutes. After the culture for another 4 hours, calcein AM and propidium iodide dyes were used for staining MCF-7 cells based on the manufacturer' s manual.

Hemolysis Assay: The blood samples were sampled from BALB/c mice and centrifuged (3000 rpm, 4 °C) for 10 minutes to collect red blood cells (RBCs). The RBCs suspended in PBS were mixed with fd-AR-TN@PtNE/ICG samples with various (0, 3.125, 6.25, 12.5, 25, 50, 100, and 200 μg mL^{-1}) concentrations. RBCs suspended in the ultrapure water were used as the hematolytic positive control. The mixtures were incubated for 2 hours at 37 °C and centrifuged (12000 rpm, 4 °C) for 5 minutes to separate the supernatants. The hemolysis ratio was determined by the absorbance of supernatants at 541 nm.

Blood routine and blood biochemical analyses: The BALB/c mice were intravenously injected with the fd-AR-TN@PtNE/ICG (100 μL, 200 μg mL^{-1}) solutions in PBS. The blood samples of mice were collected at day 8 and day 16 after injection. The common indices of the blood routine were evaluated, such as red blood cells (RBC), white blood cells (WBC), hemoglobin (HGB), platelets (PLT), mean corpuscular volume (MCV), hematocrit (HCT), mean corpuscular hemoglobin (MCH), mean corpuscular hemoglobin concentration (MCHC) and mean platelet volume (MPV). The representative hepatic and kidney function markers for blood biochemical analysis were measured, including aspartate aminotransferase (AST), alanine transaminase (ALT), blood urea nitrogen (BUN), creatinine (CREA), uric acid (UA), and alkaline phosphatase (ALP). The mice treated by PBS were used as the control group.

In vivo MCF-7 tumor-homing ability: MCF-7 tumor cells (5×10^6) were injected subcutaneously into the back of BALB/c nude mice (3-5 weeks old, female) to establish the tumor model. Once the average tumor size reached 100 mm^3, the fd-AR-TN@PtNE/ICG and fd-GE-TN@PtNE/ICG (100 μL, 200 μg mL^{-1}) solutions in PBS were intravenously injected to the mice for *in vivo* imaging studies. At the 6, 12, 24, 48, 72, 96 hours post injection, the mice were imaged by IVIS Spectrum.

Photothermal Imaging of the nanofibers in vivo: The sample solutions of fd-AR-TN@PtNE/ICG (100 μL, 200 μg mL^{-1}) were injected into the MCF-7 tumor-bearing mice. After 12 hours, the tumor sites were irradiated by the 808 nm light (0.8 W cm^{-2}) for 10 minutes. Infrared thermal images were obtained through an Infrared Thermal Imaging Spectrometer every 2 minutes. The PBS group and the ICG, fd-AR-TN@ICG groups with the same ICG concentrations were also imaged.

HIF-1α Staining: The sample solutions of fd-AR-TN@PtNE/ICG (100 μL, 200 μg mL^{-1}) in PBS were injected intravenously into the tumor-bearing mice. After 24 hours, the mice were sacrificed and the tumors were sectioned for immunofluorescence staining. The collected slices were stained by rabbit anti-HIF-1α primary antibody

overnight at 4 °C. Then, the tumor slices were stained with Cy3-conjugated anti-rabbit IgG (H+L) antibody for 1 h at 37 °C. The cell nuclei were finally stained by DAPI for 5 minutes at 25 °C.

In vivo PDT: 25 tumor-bearing mice were divided into five groups (n = 5) to carry out different treatments including PBS, ICG + NIR, fd-AR-TN@ICG + NIR, fd-AR-TN@PtNE/ICG, and fd-AR-TN@PtNE/ICG + NIR. The fd-AR-TN@PtNE/ICG nanofibers were suspended in PBS with the concentration of 200 μg mL^{-1}. The ICG concentrations in the ICG-bearing groups (ICG, fd-AR-TN@ICG, fd-AR-TN@PtNE/ICG) were identical. All groups were intravenously injected into the mice with the dose of 100 microliters. Twelve hours post injection, the groups indicated were irradiated with the 808 nm light (0.8 W cm^{-2}) for 10 minutes. The mice weight and tumor size were measured every other day since the beginning of treatments. The volume of tumors was calculated to be: $V = a^2 \times b/2$. The a and b were the shortest and longest sizes of tumors, respectively. The relative tumor volume (V/V_0) was normalized to initial tumor volume (V_0) before the treatments. The mice were sacrificed 16 days post the treatments and the tumors were weighed and collected. Major organs including liver, heart, kidney, spleen and lung of mice were also collected for studying the possible histological toxicity after the tumor therapy.

ROS staining: MCF-7 tumor-bearing mice were intravenously injected with different sample solutions. After 12 hours, DCFH-DA solutions were administrated through intraperitoneal injection (0.6 mg per mouse), followed with irradiation. The tumor tissues were collected 0.5 hour later and then frozen sections were prepared. The cell nuclei were stained by DAPI. The fluorescent images were recorded with an inverted fluorescent microscope (NIKON ECLIPSE TI-SR).

Pathological analysis: The tumor tissues collected were fixed with paraformaldehyde and then embedded with paraffin. The tissues were cut into slices and stained by H&E

to perform the histological analysis. For TUNEL staining, the tumor slices were stained by a specialized detection kit (Roche) and colored with 3,3'-diaminobenzidine (DAB). For Ki67 staining, the tumor slices were stained by rabbit anti-Ki67 monoclonal antibody (Abcam) and colored with DAB before imaging under the microscope. All the histological and immunohistochemical staining were conducted according to the standard processes.

Statistical analysis: All results were presented as the mean ± standard error of the mean. Significant difference was analyzed via one-way ANOVA method. A p-value below 0.05 was considered as significantly different (*$p < 0.05$, **$p < 0.01$, ***$p < 0.001$, ****$p < 0.0001$).

Table S1. The design of annealed oligonucleotides encoding Pt-binding peptide TN inserted into *gene VIII* or tumor-homing peptide AR inserted into *gene III* of fd phage.

Peptide sequence	Annealed oligonucleotide sequence
**TLTTLTN (TN)**	**TN Forward:** 5´-AGCTTTGCCACTCTTACGACGCTTACGAATGGTGCA-3´
	**TN Reverse:** 5´-CCATTCGTAAGCGTCGTAAGAGTGGCAA-3´
**AREYGTRFSLIGGYR (AR)**	**AR Forward:** 5´-TTGCGCGGGAGTATGGTACGAGGTTTTCTCTTATTGGTGGTTATCGGGCTG-3´
	**AR Reverse:** 5´-CCCGATAACCACCAATAAGAGAAAACCTCGTACCATACTCCCGCGCAACGT-3´

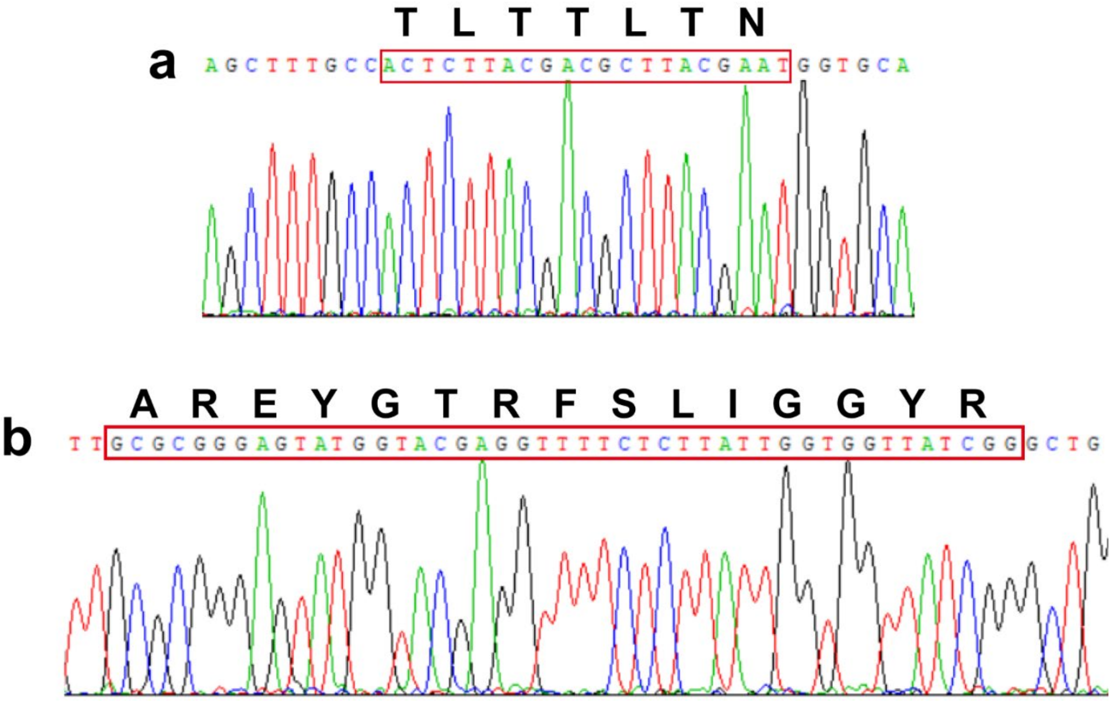

Figure S1. Results of DNA sequencing on the double-displayed fd-AR-TN phage. a) A DNA fragment encoding the TN peptide was inserted into *gene VIII* of the fd phage successfully. b) A DNA fragment encoding AR peptide was inserted into *gene III* of the fd phage successfully.

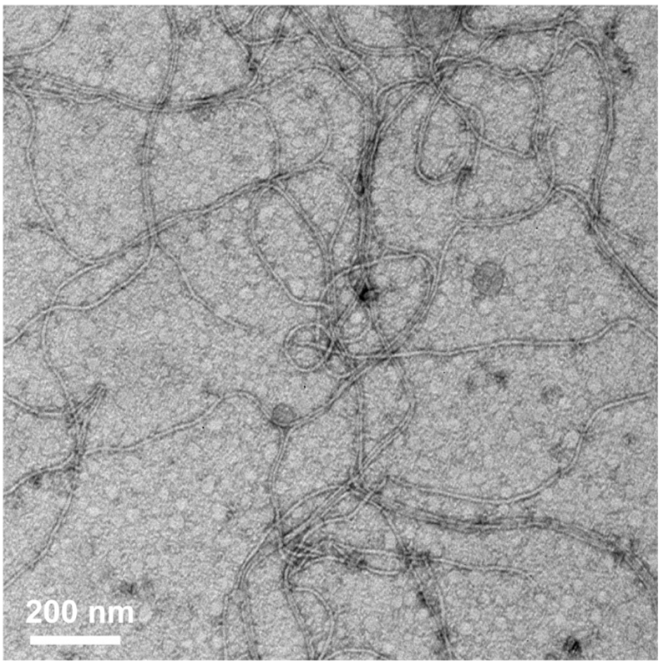

Figure S2. TEM images of the double-displayed fd-AR-TN phage.

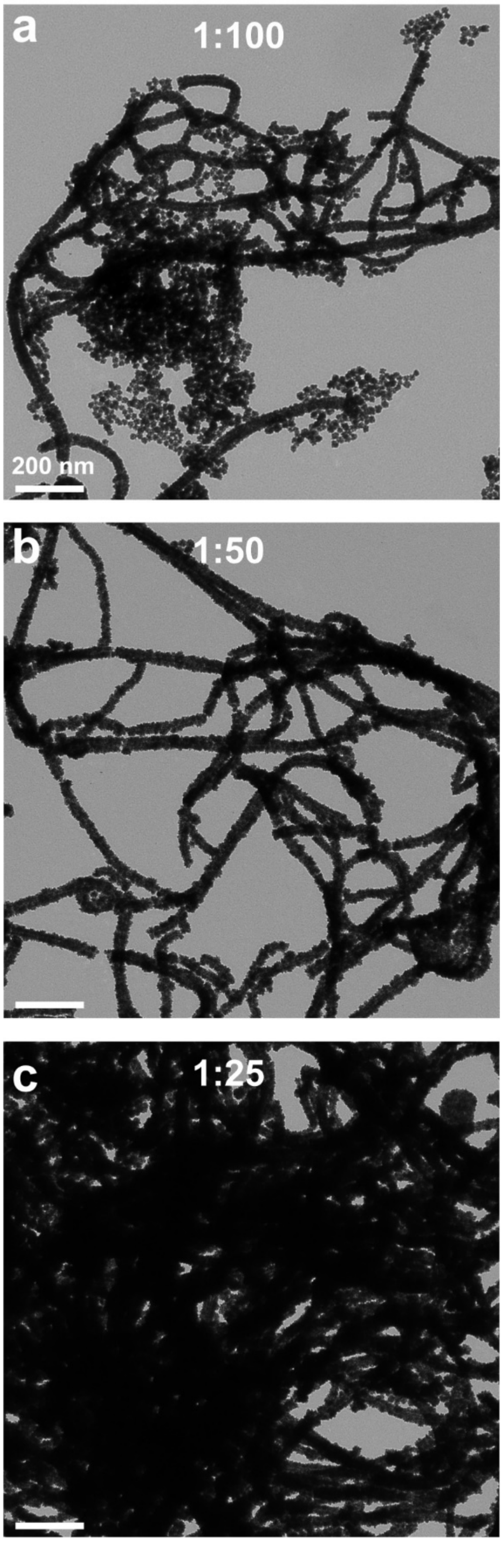

Figure S3. Phage concentration-dependent synthesis of PtNEs. The typical TEM images of fd-AR-TN@PtNE when weight ratio of phage: Pt(IV) was a) 1:100, b) 1:50 and c) 1:25. The concentration of Pt(IV) was fixed to be 2 mg/mL.

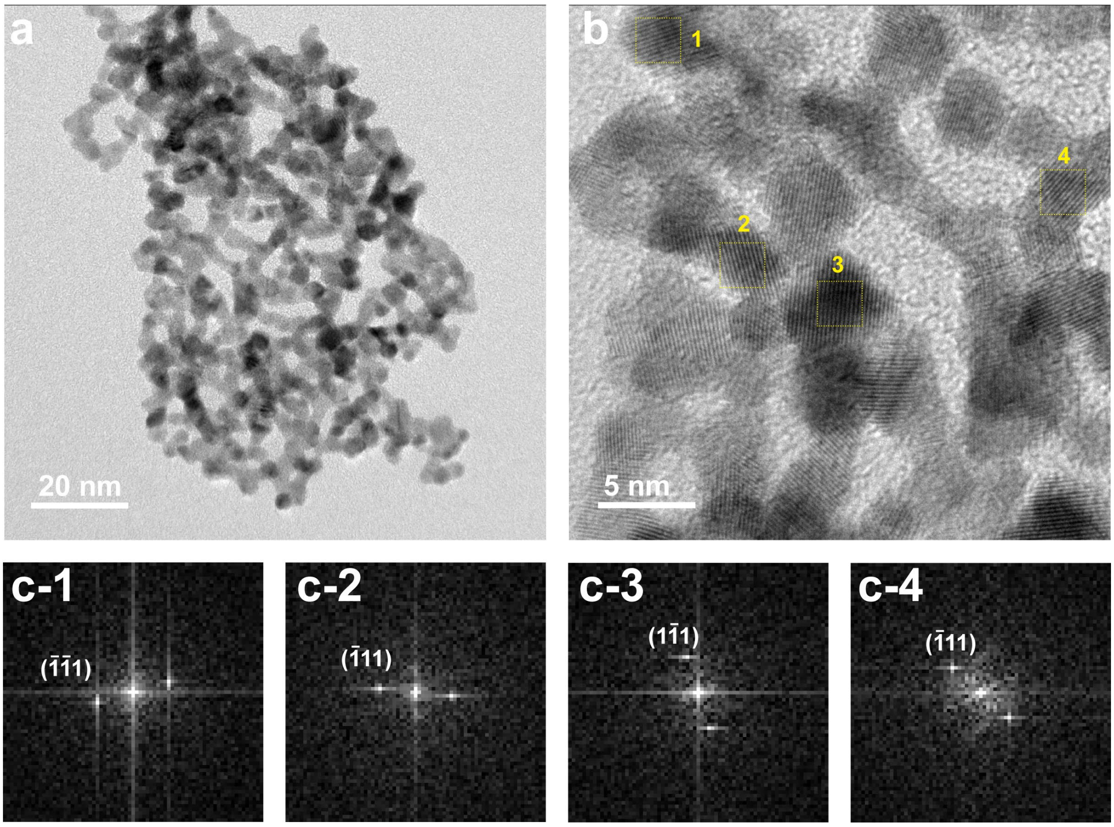

Figure S4. Synthesis and characterization of non-phage-templated PtNE. a) TEM images showing the morphology of PtNE. b) HRTEM lattice image of PtNE and four typical areas (yellow rectangles) were selected to perform the Fast Fourier transformation (FFT). c) FFT of the selected nanocrystals in the HRTEM lattice image. The numbers (c-1, c-2, c-3, c-4) correspond to the rectangles (1, 2, 3, 4) in (b) one by one.

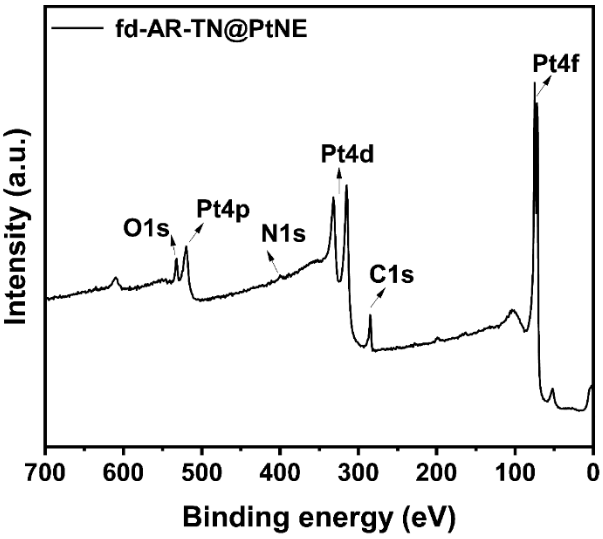

Figure S5. XPS spectrum of fd-AR-TN@PtNE nanofibers.

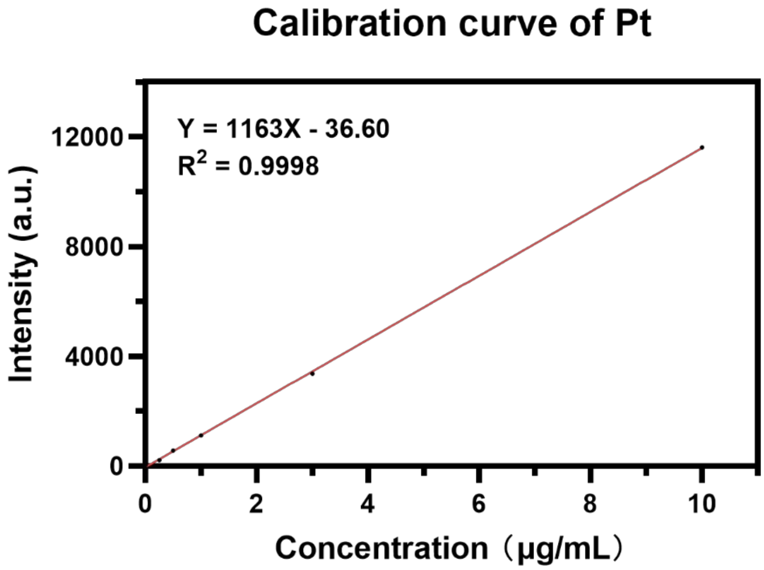

Figure S6. Standard curve of ICP-OES measurements at different concentrations of platinum standards. The standards of platinum were plotted against the concentrations based on their ICP-OES emission intensities. The platinum contents of fd-AR-TN@PtNE were calculated from their measured intensities. The standard curve presents a linear fit (red line) with $R^2 = 0.9998$.

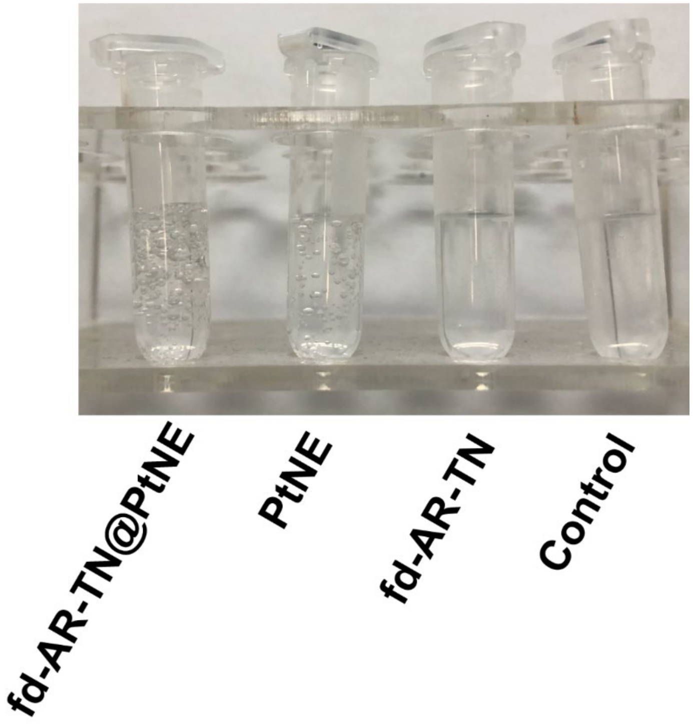

Figure S7. Photographs of H_2O_2 solutions after different treatments as indicated.

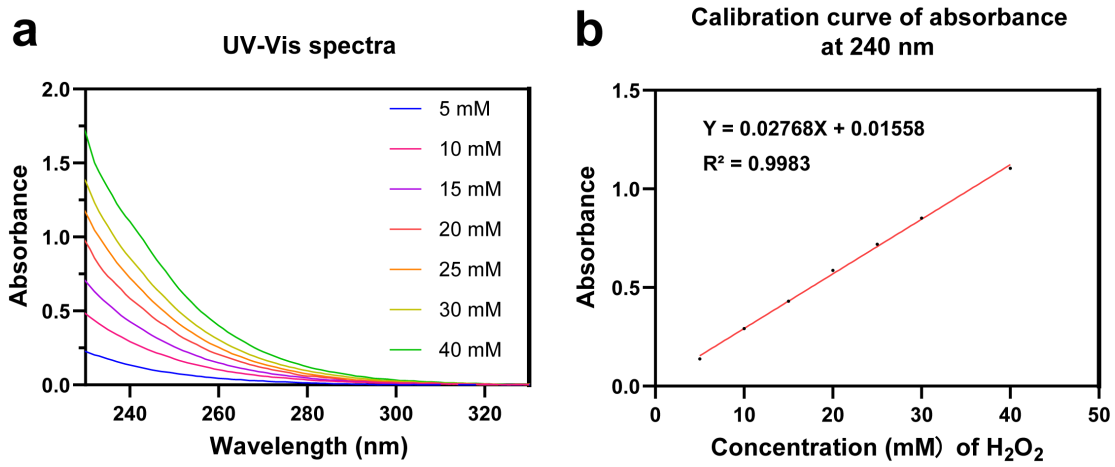

Figure S8. a) UV-Vis spectra at different concentrations of H_2O_2 standards. b) The standard curve showing the absorbance of H_2O_2 plotted against the concentrations (5, 10, 15, 20, 25, 30, 40 mM) based on their UV-Vis absorbance at 240 nm. The standard curve presents a linear fit (red line) with $R^2 = 0.9983$.

Table S2. Calculated adsorption energy values of the chemisorption of O* with different adsorption sites, including the top, bridge and hollow sites on Pt-{111} and Pt-{100}.

Pt-{111}		Pt-{100}	
Adsorption site	Adsorption energy, eV	Adsorption site	Adsorption energy, eV
Top	-0.18	Top	-0.52
Bridge	-0.81	Bridge	-1.61
Hollow	-1.11	Hollow	-0.79

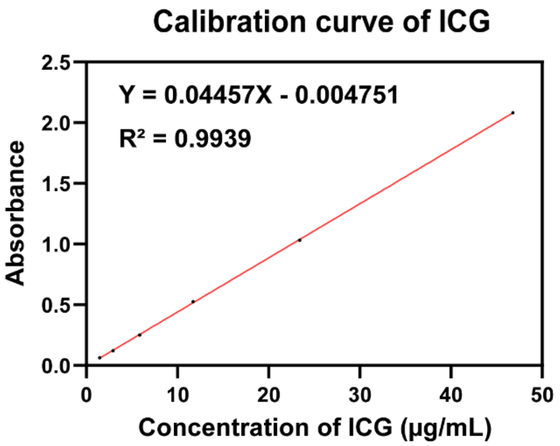

Figure S9. Standard curve of the absorption spectra measurements at different concentrations of ICG standards. The standards of ICG were plotted against the concentrations based on their absorbance intensities at 785 nm. The ICG contents of fd-AR-TN@PtNE/ICG were calculated from their measured intensities. The standard curve presents a linear fit (red line) with $R^2 = 0.9939$.

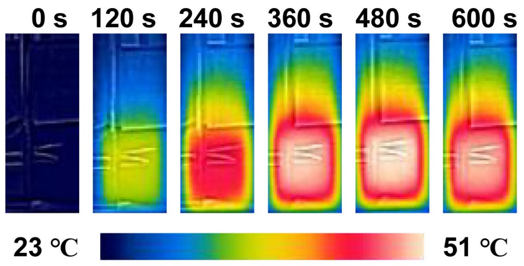

Figure S10. IR thermal images of fd-AR-TN@PtNE/ICG solutions with 15 μg/mL of ICG under 808 nm light (0.8 W cm^{-2}) irradiation.

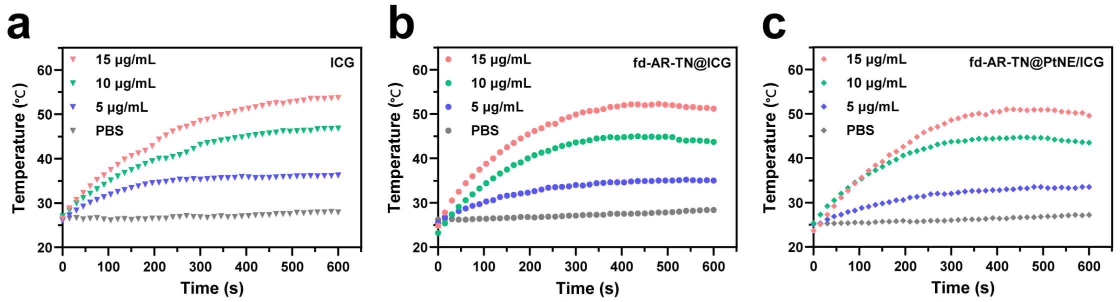

Figure S11. Temperature changes of ICG group (a), fd-AR-TN@ICG group (b) and fd-AR-TN@PtNE/ICG group (c) with various concentrations of ICG (5, 10, 15 μg/mL) under NIR light irradiation. The PBS group was set to be the control.

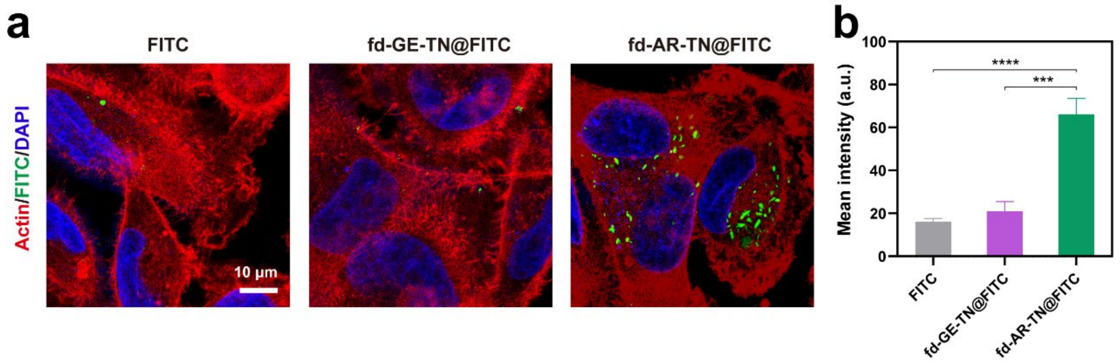

Figure S12. *In vitro* cellular uptake evaluation on MCF-7 tumor cells. a) Confocal images of MCF-7 tumor cells co-cultured with free FITC, fd-GE-TN@FITC and fd-AR-TN@FITC nanofibers. The cell nucleus was stained by DAPI (blue). b) The relative intensity analysis of green fluorescence signals from free FITC or FITC-conjugated nanofibers of various groups in (a). ***p < 0.001, ****p < 0.001.

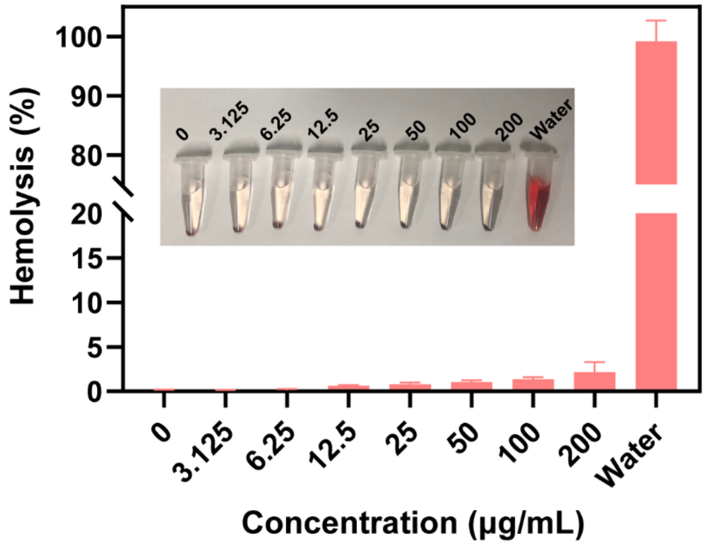

Figure S13. Hemolysis evaluation of fd-AR-TN@PtNE/ICG nanofibers solutions with different concentrations *in vitro*.

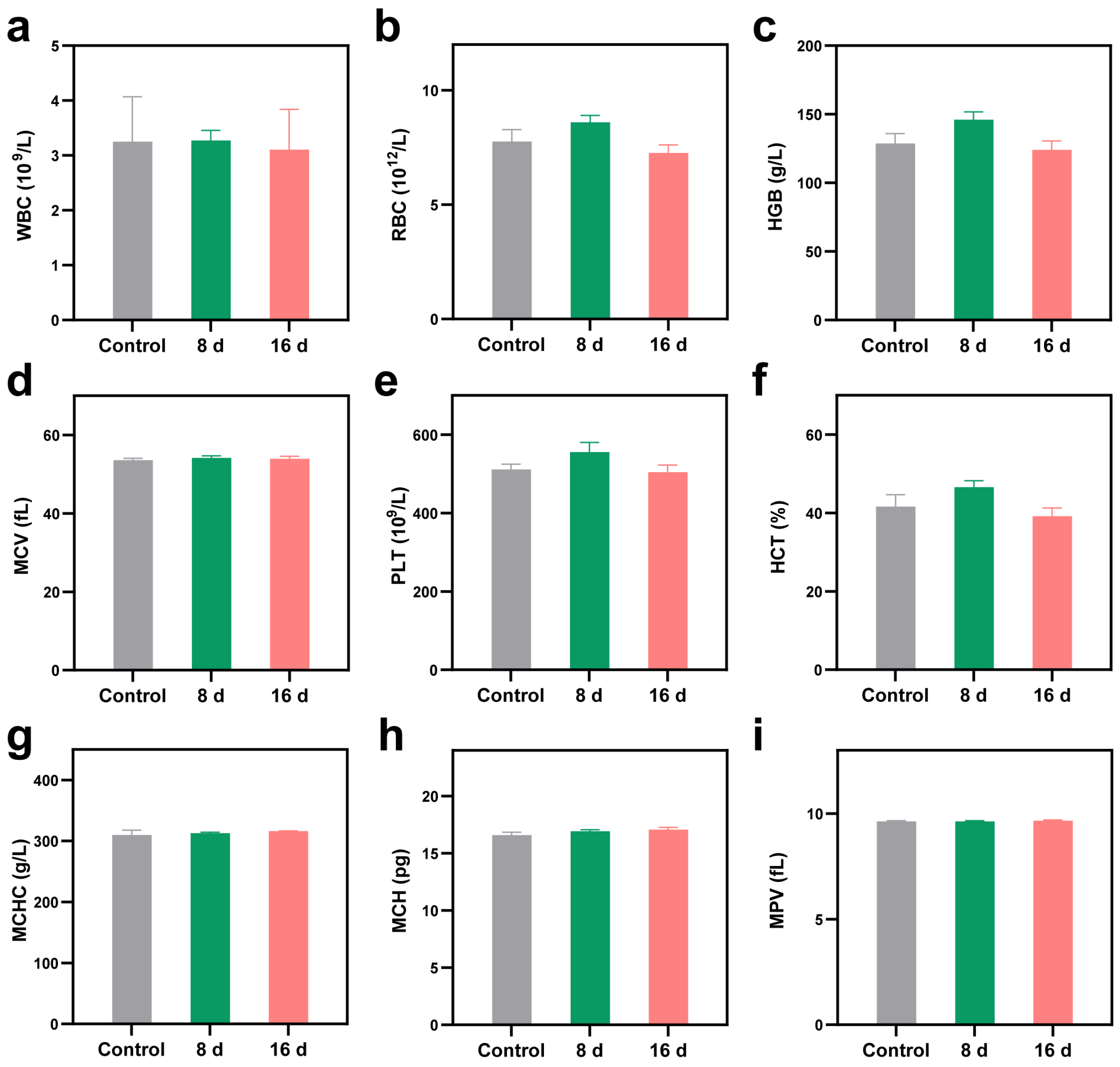

Figure S14. Blood routine analysis. Levels of WBC (white blood cells, a), RBC (red blood cells, b), HGB (hemoglobin, c), MCV (mean corpuscular volume, d), PLT (platelets, e), HCT (hematocrit, f), MCHC (mean corpuscular hemoglobin concentration, g), MCH (mean corpuscular hemoglobin, h), and MPV (mean platelet volume, i) in the mice injected with the fd-AR-TN@PtNE/ICG nanofibers at 8 d and 16 d compared with the untreated mice (control).

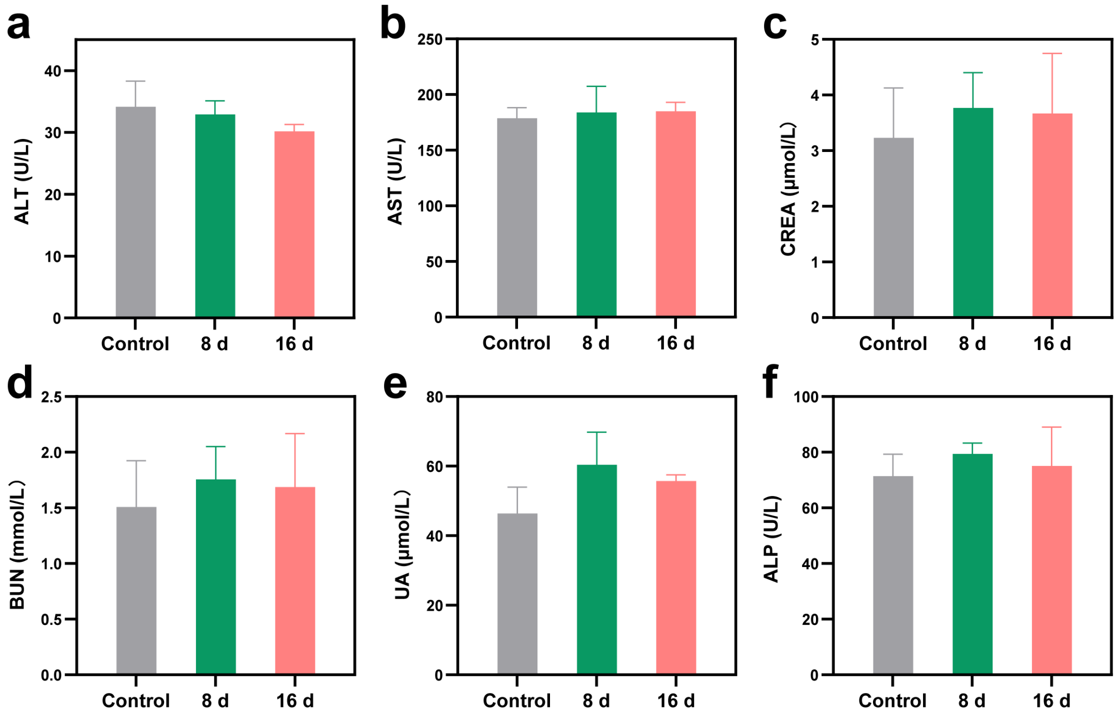

Figure S15. Blood biochemical analysis. Levels of ALT (alanine transaminase, a), AST (aspartate aminotransferase, b), CREA (creatinine, c), BUN (blood urea nitrogen, d), UA (uric acid, e) and ALP (alkaline phosphatase, f) in the mice injected with the fd-AR-TN@PtNE/ICG nanofibers on day 8 and 16 were compared with the control (untreated mice).

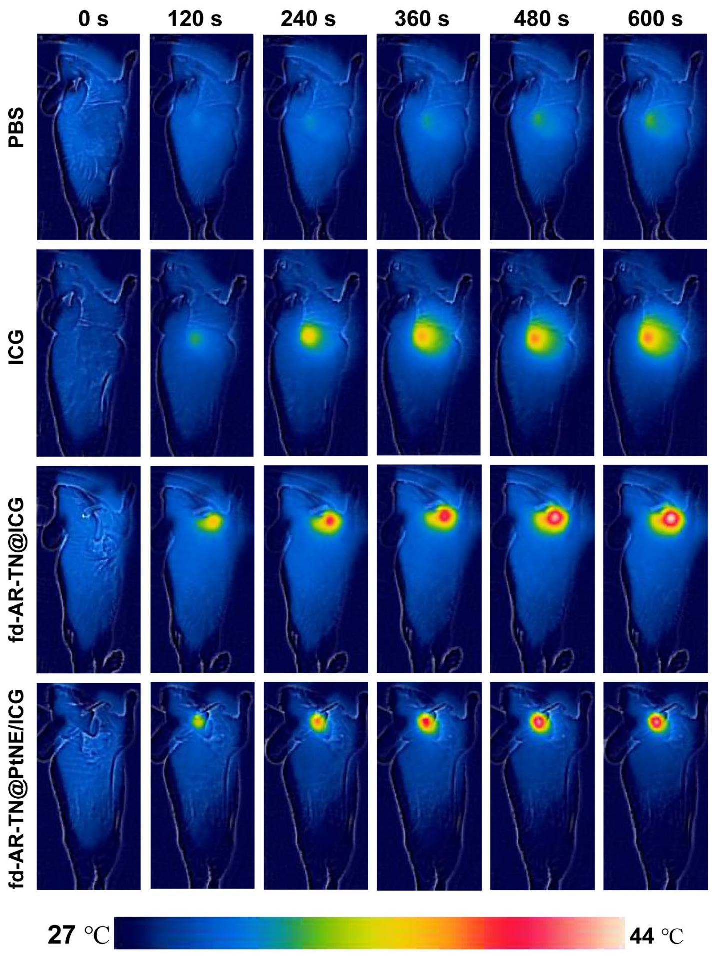

Figure S16. IR thermal images of MCF-7 tumor-bearing mice under 808 nm light irradiation 12 hours post injection with PBS, ICG, fd-AR-TN@ICG and fd-AR-TN@PtNE/ICG.

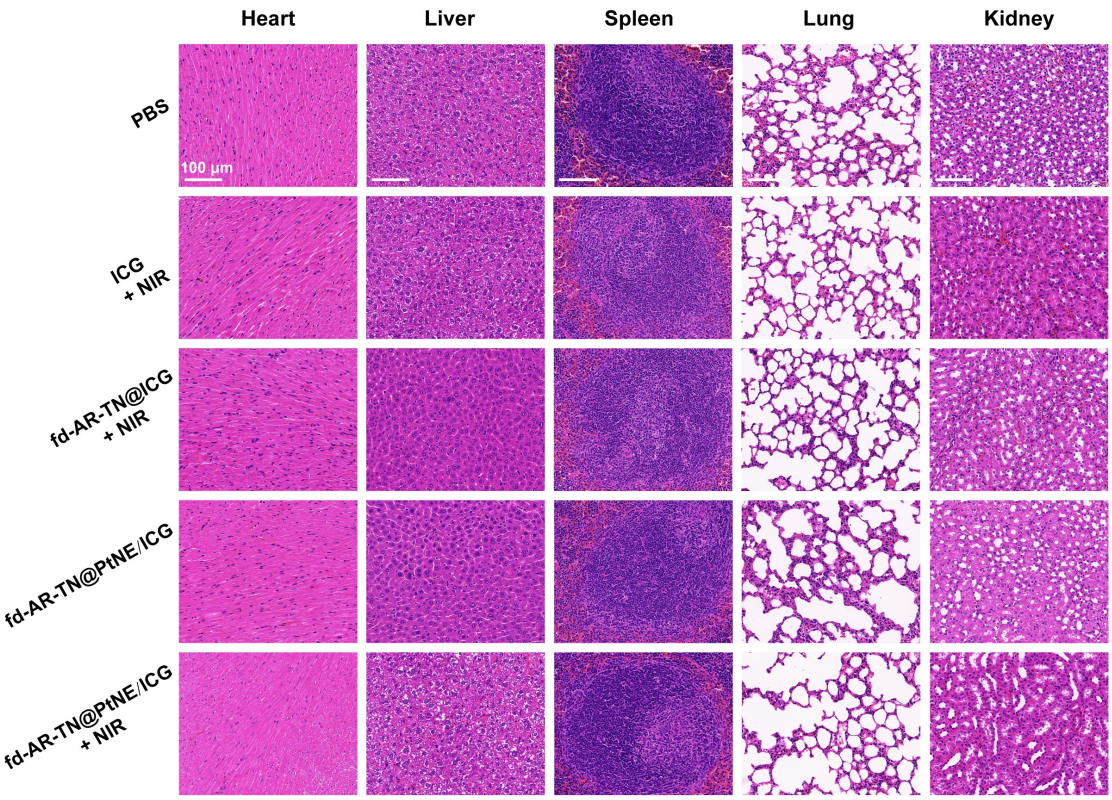

Figure S17. H&E staining images of major organs (liver, heart, kidney, lung and spleen) after various treatments indicated.

References

[1] Y. Li, X. Qu, B. Cao, T. Yang, Q. Bao, H. Yue, L. Zhang, G. Zhang, L. Wang, P. Qiu, N. Zhou, M. Yang, C. Mao, *Adv. Mater.* **2020**, 32, 2001260.

[2] G. Kresse, D. Joubert, *Physical Review B* **1999**, 59, 1758.

[3] J. P. Perdew, K. Burke, M. Ernzerhof, *Phys. Rev. Lett.* **1996**, 77, 3865.

[4] V. Wang, N. Xu, J.-C. Liu, G. Tang, W.-T. Geng, *Comput. Phys. Commun.* **2021**, 267, 108033.

[5] S. Grimme, S. Ehrlich, L. Goerigk, *J. Comput. Chem.* **2011**, 32, 1456.

[6] T. Gu, Y. Wang, Y. Lu, L. Cheng, L. Feng, H. Zhang, X. Li, G. Han, Z. Liu, *Adv. Mater.* **2019**, 31, 1806803.
